# Characterization of the Poplar R2R3-MYB Gene Family and Over-Expression of *PsnMYB108* Confers Salt Tolerance in Transgenic Tobacco

**DOI:** 10.3389/fpls.2020.571881

**Published:** 2020-10-16

**Authors:** Kai Zhao, Zihan Cheng, Qing Guo, Wenjing Yao, Huajing Liu, Boru Zhou, Tingbo Jiang

**Affiliations:** ^1^State Key Laboratory of Tree Genetics and Breeding, Northeast Forestry University, Harbin, China; ^2^Co-Innovation Center for Sustainable Forestry in Southern China/Bamboo Research Institute, Nanjing Forestry University, Nanjing, China; ^3^College of Resources and Environmental Sciences, Northeast Agricultural University, Harbin, China

**Keywords:** poplar, R2R3-MYB gene family, salt stress, Synteny analysis, Tissue-differential expression

## Abstract

The MYB, one of the largest transcription factor families in plants, is related to various biological processes. For an example, the R2R3-MYB family plays an important role in regulation of primary and secondary metabolism, plant growth and development, and responses to hormones and stresses. However, functional studies on the poplar R2R3-MYB genes are limited. In this study, we identified 207 poplar R2R3-MYB genes that are unevenly distributed on the 19 chromosomes of poplar, followed by characterization of their conserved domains. On the basis of phylogenetic analysis, these genes can be divided into 23 groups. Evidence from synteny analyses indicated that the poplar R2R3-MYB gene family is featured by tandem and segmental duplication events. On the basis of RNA-Seq data, we investigated salt responsive genes and explored their expression patterns. Furthermore, we cloned the *PsnMYB108* gene from poplar, which is significantly up-regulated in roots and leaves in response to salt stress. To validate its function, we developed transgenic tobacco plants that over-express the *PsnMYB108* gene. It appears that the transgenic lines are more tolerant to salt stress than the wild type does. Evidence from physiological analyses demonstrated that over-expression of *PsnMYB108* may improve tobacco salt stress tolerance by increasing the reactive oxygen species scavenging ability and the accumulation of proline. These results laid the foundation for future analysis and functional studies of poplar R2R3-MYB family members, and revealed that *PsnMYB108* plays an important role in improving plant salt stress tolerance.

## Introduction

The MYB gene family is widely existed in eukaryotes and has multiple functions. Since its first discovery in avian myeloblastosis virus in 1982 (named as v-myb) (Klempnauer et al., 1982), many members from the family have been identified. In plants, the first gene identified is *ZmMYBC1*, which was found in *Zea mays* in 1987 (Paz-Ares et al., 1987). Genes of the MYB family encode proteins known as transcription factors that harbor MYB domains. This domain often contains 1 to 4 incompletely repeating amino acid sequence repeats (R). Each R sequence consists of about 52 amino acids that form three alpha helices (Dubos et al., 2010). In addition, there are regularly spaced tryptophan (W) residues in each R sequence, in order to form a helix–turn–helix (HTH) structure hydrophobic core with the second and third helix (Kanei-Ishii et al., 1990; Dubos et al., 2010). Sometimes tryptophan residues are replaced by some hydrophobic amino acids. For example, the first tryptophan residue in the R3 repeat in plants is often replaced by leucine, isoleucine, or phenylalanine (Martin and Paz-Ares, 1997). There are some highly conserved amino acid sequences before and after each conserved tryptophan. The third helix of each repeat is the “recognition helix,” which directly contacts DNA and inserts into the main groove (Jia et al., 2004).

According to the numbers of the MYB repeats, the MYB family in plants can be divided into four types, namely, 4R-MYB (containing four R1/R2 repeats), 3R-MYB (containing R1, R2, R3 three repeats), R2R3-MYB (containing R2 and R3 repeats), and MYB-related (including single or a partial MYB repeat) (Dubos et al., 2010). In plants, the number of 4R-MYB and 3R-MYB genes is relatively small, and there are few studies on 4R-MYB members. Previous studies have shown that 3R-MYB genes are related to cell cycle regulation and abiotic stress responses (Feng et al., 2017). MYB-related genes are the second largest member of the MYB gene family. Genes from this subfamily have diverse functions and play an important role in plant growth and stress responses, such as cell morphogenesis, secondary metabolism, organ morphogenesis, chloroplast development, leaf senescence, response to phosphate starvation and tolerance to cold, drought and salt (Dubos et al., 2010; Li et al., 2016; Yin et al., 2017; Park et al., 2018; Sun et al., 2018).

The R2R3-MYB transcription factors are the largest subfamily of the MYB family in plants. The origin of this gene subfamily is controversial. The subfamily of genes may be derived from the 3R-MYB gene ancestor after losing the R1 repeat sequence and expanding the gene family (Rosinski and Atchley, 1998). On the contrary, studies suggested that the 3R-MYB gene was evolved from the R2R3-MYB gene by obtaining the R1 repeat (Jiang et al., 2004). Studies have indicated that the significant amplification of the R2R3-MYB proteins is mainly due to the whole genome and segmental duplication (Du et al., 2015). Its N-terminal generally has a conservative MYB domain (Dubos et al., 2010), but other areas contain extensive intrinsically disordered-regions (Millard et al., 2019). In addition to binding to specific DNA sequences, the MYB domain of the R2R3-MYB transcription factors may also have other functions, such as interacting with other transcription factors (Zimmermann et al., 2004). The intrinsically disordered-regions may be related to the functional diversity of this subfamily, due to their high sequence diversity (Millard et al., 2019). The majority of the R2R3-MYB transcription factors in plants can identify the MYB-core [C/T]NGTT[G/T] and AC-rich elements, which may contribute to DNA binding specificity (Millard et al., 2019). Previous studies have indicated that the R2R3-MYB transcription factors have multiple biological functions, such as regulation of plant growth and development and stress responses.

The R2R3-MYB transcription factor can regulate plant primary and secondary metabolism. *AtMYB12* and *AtMYB111* can control flavonoid biosynthesis in Arabidopsis (Stracke et al., 2007). Narcissus R2R3-MYB transcription factor gene *NtMYB3* can regulate flavonoid biosynthesis (Anwar et al., 2019). *FeMYBF1* regulates flavonoid biosynthesis in buckwheat (Matsui et al., 2018). Pear R2R3-MYB gene *MYB114* can interact with other transcription factors to regulate the anthocyanins biosynthesis of fruit (Yao et al., 2017). *DcMYB6* is involved in regulating anthocyanin biosynthesis in purple carrot taproots (Xu et al., 2017). Poplar transcription factors MYB115 and MYB134 regulate the synthesis of proanthocyanidins (James et al., 2017). *AtMYB46* is related to the regulation of secondary wall biosynthesis in Arabidopsis (Zhong et al., 2007). *OsMYB46* and *ZmMYB46* are the major transcriptional activators of secondary wall biosynthesis programs in rice and maize, respectively (Zhong et al., 2011). Poplar R2R3-MYB transcription factor PtrMYB152 regulates secondary cell wall biosynthesis in Arabidopsis (Wang et al., 2014). R2R3-MYB transcription factor can also regulate plant growth and development. *AtMYB108* and *AtMYB24* jointly regulate jasmonic acid-mediated stamen maturation in Arabidopsis (Mandaokar, 2009). *Artemisia annua AaMYB1* is associated with trichome development (Matías-Hernández et al., 2017). PtoMYB216 is a specific transcriptional activator of poplar lignin biosynthesis and participates in the regulation of poplar wood formation (Tian et al., 2013). The R2R3-MYB transcription factor can also participate in the response to plant hormones. In *Arabidopsis thaliana*, *MYB96* regulates the positive and negative regulators of ABA signaling through a unique molecular mechanism, thereby conferring maximum sensitivity on ABA (Lee and Seo, 2019). Rice *OsMYB103L* plays an important role in GA-regulated secondary cell wall synthesis (Ye et al., 2015). *AtMYB30* participates in the amplification loop or signaling cascade that regulates SA synthesis, which in turn regulates cell death (Raffaele et al., 2006). Previous studies have shown that some Arabidopsis R2R3-MYB transcription factor genes are induced by ABA, IAA and cytokinin (Kranz et al., 1998), which suggests that the R2R3-MYB transcription factor in Arabidopsis may be widely involved in the response to plant hormones. The R2R3-MYB transcription factor can also respond to biotic and abiotic stresses. For example, the Arabidopsis R2R3-MYB transcription factor BOS1 can affect the tolerance of plants to biotic and abiotic stresses (Mengiste et al., 2003). The R2R3-MYB gene *AtMYB30* is a positive regulator of the hypersensitive cell death program in plants responding to pathogen invasion (Vailleau et al., 2002). Sweet cherry R2R3-MYB transcription factor PacMYBA is a positive regulator of salt stress tolerance and pathogen resistance (Shen et al., 2017). According to previous reports, plant R2R3-MYB transcription factors can respond to abiotic stresses such as drought stress, cold stress, salt stress, osmotic stress, phosphate starvation, etc. (He et al., 2016).

The R2R3-MYB genes play an important role in plant growth and development, and previous studies have shown that many R2R3-MYB genes in plants can respond to salt stress. However, previous studies have only focused on the role of poplar R2R3-MYB transcription factor in the regulation of synthesis of anthocyanins, lignin and secondary walls. Studies on R2R3-MYB genes in response to salt stress are limited in poplar. In this study, we identified 207 poplar R2R3-MYB transcription factor genes. Subsequently, a comprehensive analysis including phylogenetic analysis, chromosome distribution, gene duplication, and synteny analysis was performed. Based on the analysis of transcriptome data, we also explored the tissue differential expression patterns and the response to salt stress of these genes. Then we cloned an up-regulated gene under salt stress, *PsnMYB108*. Then, quantitative real-time PCR (qRT-PCR) was used to further analyze the tissue differential expression and the spatiotemporal expression pattern under salt stress. The results of particle bombardment transient transformation experiments showed that PsnMYB108 protein was localized in the nucleus. Through *Agrobacterium tumefaciens*-mediated transformation, we successfully obtained transgenic tobacco plants that overexpress the *PsnMYB108* gene. The experimental results of salt stress tolerance and physiological indexes showed that overexpression of *PsnMYB108* gene in tobacco can improve the plant’s ability of salt stress tolerance.

## Materials and Methods

### Identification of the R2R3 MYB Family Members and Conserved Protein Domains in Poplar

The identification of R2R3-MYB gene family in poplar was performed according to a previous study (Chen et al., 2019). We downloaded the amino acid sequence of poplar protein from the Phytozome database (Tuskan et al., 2006; Goodstein et al., 2012). Then we used the Myb_DNA-binding (PF00249) downloaded from the Pfam database to search the amino acid sequence (E-value < 1 × 10^–5^) (El-Gebali et al., 2019). After obtaining the candidate genes, we screened out the members containing two MYB repeats, and then used Pfam and SMART databases for further verification (Letunic et al., 2015; Letunic and Bork, 2018; El-Gebali et al., 2019). After identifying the members of the R2R3-MYB gene family, we extracted the amino acid sequences of R2 and R3 MYB repeats, respectively, and used ClustalX 1.83 for multiple sequence alignment (Thompson et al., 1997). The alignment result was visualized using WebLogo and BioEdit (Schneider and Stephens, 1990; Hall, 1999; Crooks et al., 2004).

### Phylogenetic Trees

In order to explore the evolutionary relationship between the members of the poplar R2R3-MYB proteins, we constructed the phylogenetic tree, using the amino acid sequences of the R2R3-MYB family from both Arabidopsis and poplar. The Arabidopsis R2R3-MYB family members are based on previous studies (Dubos et al., 2010). We downloaded the sequences from the Phytozome database (Tuskan et al., 2006; Goodstein et al., 2012; Lamesch et al., 2012). Then we used ClustalX 1.83 for multiple sequence alignment (Thompson et al., 1997). The phylogenetic tree was constructed, using MEGA5.05 with the neighbor-joining method, 1000 repetitions of bootstrap tests, and the Poisson model (Tamura et al., 2011). Then we classified the R2R3-MYB family members of poplar, according to the previous research in Arabidopsis (Dubos et al., 2010).

### Chromosome Distribution and Gene Duplication

In order to determine chromosomal distribution of poplar R2R3-MYB gene family, we downloaded the annotation information of the poplar genome from the Phytozome database and visualized the gene distribution using TBtools (Tuskan et al., 2006; Goodstein et al., 2012; Chen et al., 2020). We use the TBtools, with Multiple Collinearity Scan toolkit (MCScanX) and BLASTP method, to analyze gene duplication events and their synteny relationship across species (Wang et al., 2012; Chen et al., 2020). Non-synonymous (ka) and synonymous (ks) substitutions of identified gene pairs were also calculated using TBtools (Chen et al., 2020).

### Gene Expression Analysis

In order to characterize expression patterns of the poplar R2R3-MYB gene family in response to salt stress and gene expression differences across tissues, we analyzed our RNA-Seq data from previous studies (Zhao et al., 2018). These data contains the gene expression in the leaves, stems, and roots of *Populus simonii* × *Populus nigra* treated with 0 or 150 mM NaCl for 24 h. The RNA-Seq data has approximately tenfold sequencing depths and high correlation between biological repeats (Zhao et al., 2018, 2019b). Then we use the DESeq package in R to identify differentially expressed genes (DEGs), the threshold is fold change ≥ 2 and padj (*p*-value adjusted for multiple testing) ≤ 0.05 (Anders and Huber, 2010).

### Experimental Materials

The poplar material used is di-haploid *Populus simonii* × *Populus nigra*, grown on 1/2 MS solid medium containing 20 g/L sucrose. The tobacco material is *Nicotiana tabacum L. cv. Petit Havana SR-1*, grown on 1/2 MS solid medium containing 20 g/L sucrose, 0.05 mg/L NAA, 0.05 mg/L IBA. The differentiation medium is MS solid medium with 2 mg/L 6-BA + 0.1 mg/L NAA + 30 g/L sucrose. The growth conditions of the culture room are 60% to 70% relative humidity, 16 h light/8 h dark, and an averaged temperature of 25°C.

### Gene Cloning and Vector Construction

Based on the transcript sequence of *PsnMYB108 (Potri.010G149900.1)*, we designed the primers required for the experiment (Tuskan et al., 2006; Goodstein et al., 2012). One-month-old tissue cultured *Populus simonii* × *Populus nigra* was quickly frozen in liquid nitrogen for RNA extraction. The detailed steps of vector construction refer to our previous research (Zhao et al., 2019a). The primers and restriction sites used for cloning genes and constructing vectors are shown in Supplementary Table S1.

### Spatiotemporal Expression Patterns of *PsnMYB108* Genes

We used qRT-PCR to analyze the expression patterns of *PsnMYB108* gene. One-month-old tissue culture seedlings were planted in the soil and grew for 1 month in the greenhouse. The growth conditions are the same as the tissue culture room.

Then we divided all the experimental materials into six groups and treated them with 150 mM NaCl for 0, 3, 6, 9, 12, and 24 h, respectively. After the treatment, samples were harvested at the same time, including leaf, stem and root tissues. The samples were quickly frozen in liquid nitrogen. All samples have three biological replicates. Primer sequences are shown in Supplementary Table S1. Actin was used as reference gene (Regier and Frey, 2010). Detailed experimental steps refer to our previous study (Yao et al., 2016).

### Subcellular Localization of PsnMYB108

The subcellular localization of PsnMYB108 protein was performed according to the previous study (Wang et al., 2019a, b). The coding sequence of *PsnMYB108* gene without termination codon was linked to the 5’ terminus of the *GFP* gene, in order to construct the pBI121-PsnMYB108-GFP fusion expression vector. The primers and restriction sites used to construct the vector are listed in Supplementary Table S1. Then we transferred the gene fusion expression vector (pBI121-PsnMYB108-GFP) and the control vector (pBI121-GFP) into the onion epidermal cells using particle bombardment (Bio-Rad, Hercules, CA, United States). The bombarded onion epidermises were cultured in the dark for 24–36 h, then they were observed under the confocal laser scanning microscope (LSM 700, Zeiss, Germany) after staining with 100 ng/mL DAPI in the dark for 15 min (staining the nucleus).

### Generation and Identification of Transgenic Tobacco

In order to obtain transgenic tobacco plants, we transformed the pROK II-PsnMYB108 plant overexpression vector into Agrobacterium and then cultivated the transgenic tobacco plants using *Agrobacterium tumefaciens*-mediated transformation (Gallois and Marinho, 1995). Resistant shoots that can grow normally on rooting medium containing 100 mg/L kanamycin and 200 mg/L ceftriaxone sodium are cultivated for 1 month, and the leaves were harvested for DNA extraction. We applied the primers used to clone the gene for molecular detection. Wild-type tobacco DNA and pROK II-PsnMYB108 vector plasmid were used as negative and positive controls, respectively. Then we randomly selected three plants with successful molecular testing, and planted them in soil pots to collect T1 generation seeds. The T1 generation seeds were sterilized and sown on rooting selection medium containing 100 mg/L kanamycin and 200 mg/L ceftriaxone sodium. Normally grown seedlings were selected and planted in soil pots in the greenhouse until T2 generation seeds were collected. The same method was used to obtain T3 homozygous seeds for salt stress tolerance analysis.

### Salt Stress Tolerance Analysis

The wild-type tobacco seeds and three T3 generation homozygous seeds with *PsnMYB108* overexpressed were sterilized. Then they were sown on tobacco rooting medium containing 0 or 150 mM NaCl (30 grains each), and vernalized at 4°C for 3 days. Subsequently, the seeds grew in the tissue culture room. We observed and recorded seed germination every day after 3 days. Three independent experiments were performed.

In addition, the uniform wild-type and transgenic tobacco seedlings (1 week old after germination) are transferred to rooting medium containing 0 or 150 mM NaCl, and continue to grow in the greenhouse. After growing for 7 days, we observed and recorded the growth of tobacco roots of different lines. Three biological replicates were used.

### Measurement of Physiological Traits

The T3 generation transgenic lines and wild-type plants were transplanted into soil, and grew for 1 month in the greenhouse. The plants were subsequently treated with 150 mM NaCl for 5 days, and water treatment was used as a control. After the treatment, the leaf tissues of different plants were harvested for the determination of physiological traits. H_2_O_2_ and proline contents were measured using reagent kits (Nanjing Jiancheng Bioengineering Institute, Nanjing, China). O_2_^–^ content was measured by O_2_^–^ content assay kit (Solarbio Science and Technology, Beijing, China). SOD and POD activities were measured using the kits from Suzhou Comin Biotechnology (Suzhou, China). Measurement of malondialdehyde (MDA) content refers to the previous method (Madhava Rao and Sresty, 2000). The determination of electrolyte leakage rate followed the method of Fan (Fan et al., 1997).

### Expression Analysis of ABA-Responsive Genes

One-month-old T3 generation transgenic and wild-type tobacco plants grown in soil were treated with 0 or 150 mM NaCl for 24 h, respectively, and leaf tissues were collected for RNA extraction and reverse transcription experiments. The qRT-PCR experiment was used to analyze the relative expression levels of tobacco ABA-responsive genes (*ABF2*, *RD29B*, *Rd22*). *Tubulin* was used as reference gene.

## Results

### Identification of R2R3 MYB Family Members and of Their Conserved Protein Domains in Poplar

We first removed redundant sequences and screened the 358 proteins containing the MYB repeats, using the HMMER analysis. Then we screened out the proteins harboring only two MYB repeats, followed by verification with evidence from the Pfam and SMART (Letunic et al., 2015; Letunic and Bork, 2018; El-Gebali et al., 2019). Finally, we identified 207 poplar R2R3 MYB family members. We then extracted the amino acid sequences of R2 and R3 MYB repeats from the conserved domain of each member, and performed multiple sequence alignment and visualization (Schneider and Stephens, 1990; Thompson et al., 1997; Hall, 1999; Crooks et al., 2004). The result of multiple sequence alignment of the MYB repeat is shown in Supplementary Figure S1. Poplar R2R3 MYB family members’ R2 and R3 MYB repeats alignment logo and annotation information are shown in Supplementary Figure S2. It appears that the MYB repeat is the same as those represented in other species (Dubos et al., 2010). The R2 and R3 MYB repeats in poplar also contain three α-helices and three conservative tryptophan (W). The first tryptophan can be replaced by some hydrophobic amino acids, such as phenylalanine (F), isoleucine (I), and leucine (L) in the R3 MYB repeat. In addition, we also found that there are many conserved sites in the R2 and R3 MYB repeats, especially at their C-terminal, which may function as recognition and binding sites of downstream genes.

### Phylogenetic Tree, Chromosome Location, and Synteny Analysis

**FIGURE 1:**
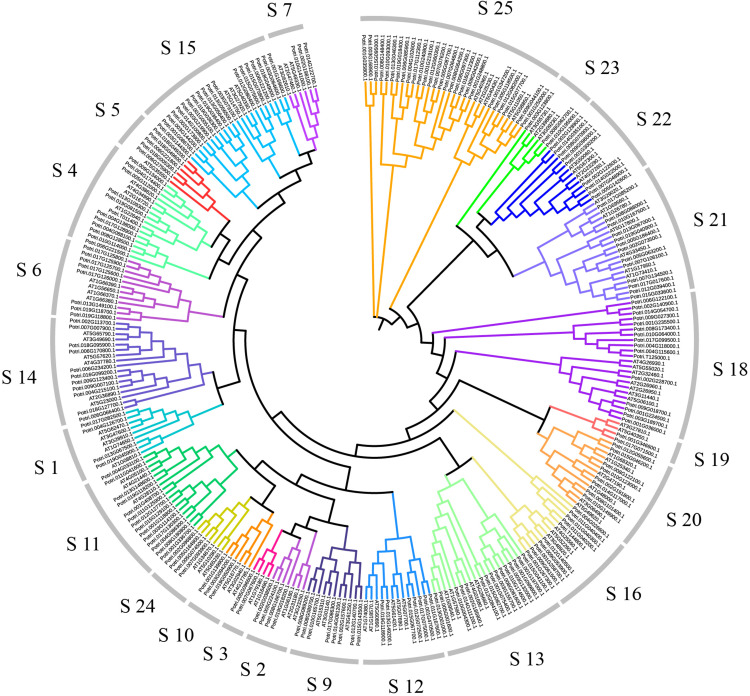
Dendrogram of the R2R3-MYB gene family proteins of poplar and Arabidopsis. The dendrogram was constructed by MEGA5.05 with the neighbor-joining method and Poisson model. Each color represents a special class.

In order to explore relationships between the R2R3-MYB proteins, we constructed the NJ-phylogenetic tree, using the amino acid sequences from both Arabidopsis and poplar. The results are shown in Supplementary Figure S3. According to the classification in Arabidopsis, we divided the poplar R2R3-MYB proteins into 23 groups (Figure 1). The average size of these groups is 9, ranging from 2 to 27.

**FIGURE 2:**
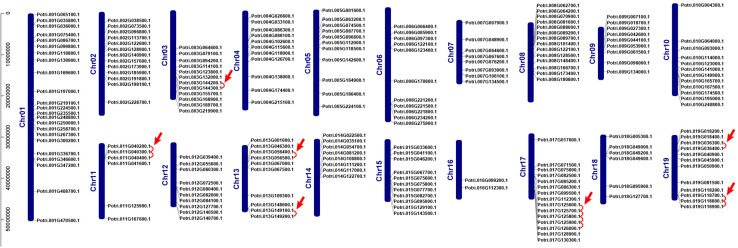
Chromosome distribution and tandem duplication events of poplar R2R3-MYB gene. Chr01–Chr19 are abbreviations for chromosome numbers 01–19. Red arrows and lines refer to gene pairs of tandem duplication.

We investigated chromosomal distribution of the poplar R2R3 MYB gene family, using the poplar genome annotation and the TBtools for visualization (Tuskan et al., 2006; Goodstein et al., 2012; Chen et al., 2020). As shown in Figure 2, the 204 R2R3 MYB genes are unevenly distributed over the 19 chromosomes of poplar. Three genes are distributed on scaffold 25, 221, and 408. In general, the number of genes on each chromosome is irrelevant to the size of the chromosome, although the largest chromosome 1 contains the most genes (23) and a smaller chromosome 16 harbors the least (2). For example, the smallest chromosome 9 has 9 genes, but chromosome 5 with a size twice of chromosome 9 has only 11 genes.

**FIGURE 3:**
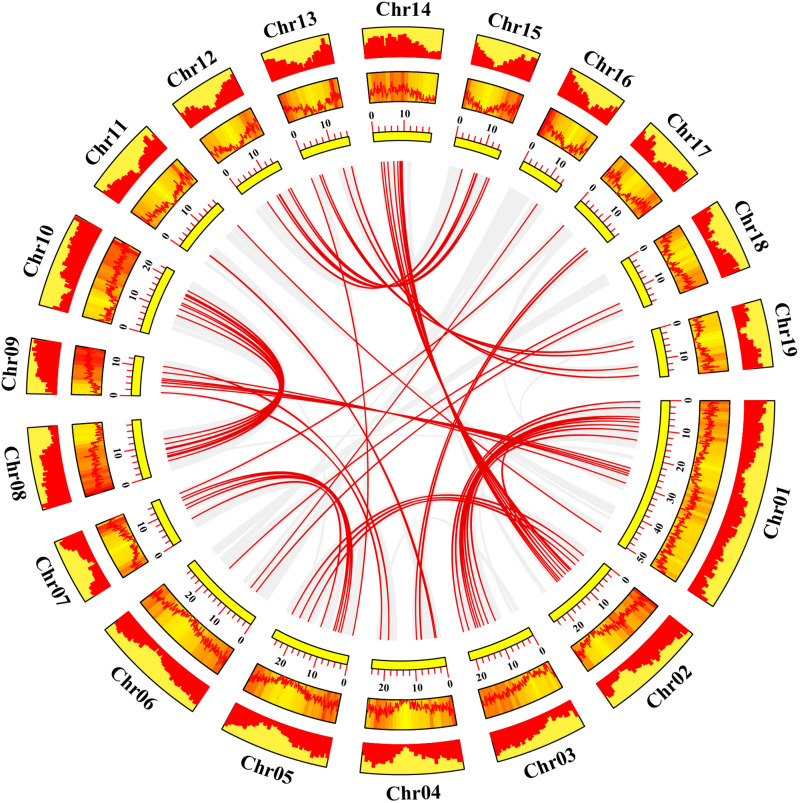
Collinearity analysis of poplar R2R3-MYB genes. Yellow rectangles represent chromosomes 01–19 of poplar. The line graph, heatmap and histogram in the orange rectangles represent the gene density on the chromosomes. Gray lines indicate all synteny blocks in the poplar genome, and the red lines between chromosomes represent the segmental duplicate gene pairs.

Gene duplication events play essential roles in formation of gene family (Gang et al., 2019). Using TBtools and MCScanX methods, we examined the poplar R2R3 MYB genes for tandem duplication events (Wang et al., 2012; Chen et al., 2020). As shown in Figure 2 and Supplementary Table S2, a total of 19 genes from chromosome 3, 11, 13, 17, and 19 are classified into 12 tandem duplication events. Among them, chromosome 17 has the most tandem duplication events (4) and the events occur in the same region on the chromosome. In addition, we also identified segmental duplication events, using TBtools with BLASTP and MCScanX methods (Wang et al., 2012; Chen et al., 2020). As shown in Figure 3 and Supplementary Table S2, a total of 64 gene pairs were observed to have segmental duplication events, among 126 genes that are distributed on 19 chromosomes. These results indicated that at least a portion of the poplar R2R3-MYB genes might be derived from gene duplication, in which segmental duplications might be a driving force for the events.

**FIGURE 4:**
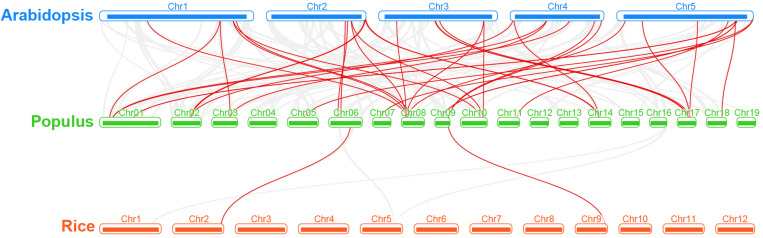
Synteny analysis of R2R3-MYB genes between poplar and two representative plant species. Gray lines indicate the collinear blocks within poplar and other plant genomes. The red lines highlight the syntenic R2R3-MYB gene pairs.

Regarding the poplar R2R3-MYB genes, we constructed a syntenic map by comparisons of sequence similarity between poplar and two representative species, Arabidopsis (dicot) and rice (monocot) (Figure 4). A total of 32 poplar genes have collinear relationships with 27 Arabidopsis genes and 2 rice genes (Supplementary Table S3). The number of orthologous pairs between poplar and Arabidopsis is 38, which is far more than the number between poplar and rice (2). This might be due to the fact that Arabidopsis and poplar are both dicot. It is worth noting that we found that seven poplar R2R3-MYB genes have collinear relationships with two genes in Arabidopsis, respectively. These conserved genes might share important functions across the species. In addition, it is interesting that, the poplar-Arabidopsis gene pairs identified were not found between poplar and rice, except for the gene pair related to *Potri.009G134000.1.* The findings suggest that the orthologous genes might occur after the differentiation of monocot and dicot. However, the orthologous gene related to *Potri.009G134000.1* might exist before the differentiation.

In order to explore the evolutionary constraints on the poplar R2R3-MYB gene family, we calculated the Ka/Ks ratios of the gene pairs. The Ka/Ks ratios are less than 1 for all of the both tandemly and segmentally duplicated poplar R2R3-MYB gene pairs, as well as for the collinear gene pairs, except for some collinear gene pairs without Ks values, based on poplar-Arabidopsis or poplar-rice comparisons. These results suggest that the poplar R2R3-MYB gene family might undergo a strong purification selection pressure over the evolution process.

### Differential Gene Expression Between Tissues Without Treatment

**FIGURE 5:**
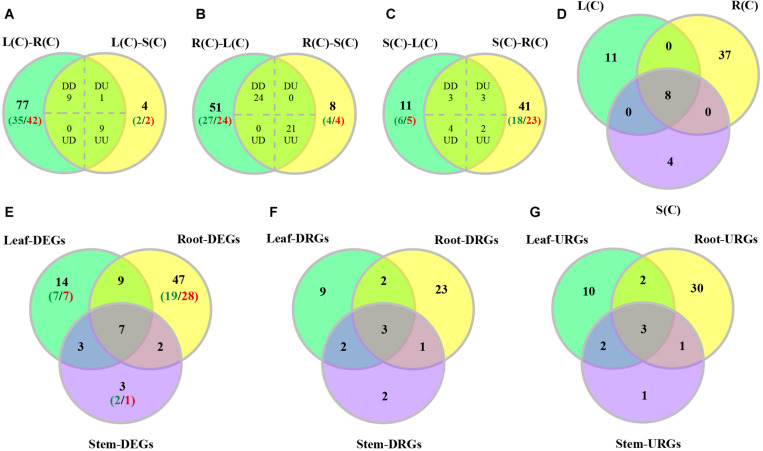
Venn diagrams of differentially expressed poplar R2R3-MYB genes over tissues and in response to salt stress. **(A–C)** Number of genes displaying distinct and shared expression in tissue pairs without treatment. L(C), leaves without treatment; S(C), stems without treatment; R(C), roots without treatment; DD, down-regulated in the two comparisons; DU, down-regulated in the left comparison and up-regulated in the right comparison; UD, up-regulated in the left comparison and down-regulated in the right comparison; UU, up-regulated in the two comparisons. **(A)** Comparison between leaf-root and leaf-stem pairs. **(B)** Comparison between root-leaf and root-stem pairs. **(C)** Comparison between stem-leaf and stem-root pairs. **(D)** Comparison among the three sets of shared genes from panels **(A–C)**. **(E–G)** Number of DEGs, down-regulated DEGs (DRGs), or up-regulated DEGs (URGs) in response to salt stress in each tissue. The green numbers indicate the number of down-regulated genes, and the red numbers indicate the number of up-regulated genes.

We characterized tissue differential expression of the R2R3-MYB genes in poplar, by pairwise comparisons including leaf-root, leaf-stem and stem-root. Results indicated that the most number (96) of DEGs were identified in leaf-root, followed by stem-root (53), and by leaf-stem (23) (Supplementary Data set S1). In addition, we investigated DEGs from one tissue relative to the other two tissues. As shown in Figures 5A–C, the majority of the DEGs (45) were observed in the compare between roots and the rest, of which 24 genes are up-regulated in roots and the remaining 21 genes were down-regulated (Supplementary Data set S1). In the comparison between leaves and the other two tissues, 19 DEGs were found, of which nine genes were up-regulated and nine genes were down-regulated. When stems were compared to the other two tissues, we detected 12 DEGs, of which only three genes were up-regulated and two genes were down-regulated (Figures 5A–C and Supplementary Data set S1). Finally, we extracted the three sets of DEGs and obtained eight shared genes (Figure 5D), indicating that these genes are differentially expressed in any tissue compared to the others.

**FIGURE 6:**
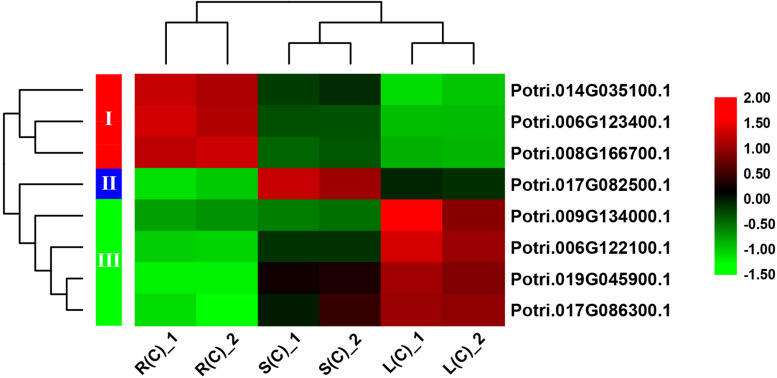
A heatmap of eight genes shared by the pairwise tissue comparisons without treatment. L(C), leaves without treatment; S(C), stems without treatment; R(C), roots without treatment. The gene expression levels are square-root transformed FPKM values. The colorful vertical bars on the left side denote gene clusters.

In order to explore the expression patterns of these eight genes across the tissues, we plotted heat maps based on the gene expression data from RNA-Seq. As expected, these eight genes display differential expression patterns (Figure 6). On the basis of cluster analysis, we divide them into three groups. The first group contains three genes that are highly, moderately and lowly expressed in roots, stems and leaves, respectively. The second group has only one gene, which is highly expressed in stems, and lowly expressed in roots. The third group has four genes with an expression pattern that is opposite to the first group (Figure 6).

### Gene Expression in Response to Salt Stress

In order to explore expression pattern of the R2R3 MYB gene family in response to salt stress in poplar, we analyzed the RNA-Seq data collected from samples with or without 24 h salt stress. We found that there were 65 DEGs in roots after salt stress (with respective 36 and 29 genes up- and down-regulated), followed by 33 DEGs in leaves (with respective 17 and 16 genes), and by 15 DEGs in stems (with respective seven and eight genes) (Supplementary Data set S2).

We then compared these DEGs across different tissues. We found that the majority of DEGs (47) were differentially expressed only in roots, followed by 14 genes only in leaves, and by 3 genes only in stems (Figure 5E). In addition to the seven genes that can respond to salt stress in all three tissues, there are nine genes responsive to salt stress in both leaves and roots, and three genes in both leaves and stems, and two genes in both stems and roots (Figure 5E).

In addition, we also compared up-regulated and down-regulated DEGs in response to salt stress across tissues. The results are shown in Figures 5F,G. Furthermore, we also stratified the DEGs by 2–4, 4–8, 8–16, 16–32, and over 32-fold, respectively. And the numbers of DEGs in each stratum are shown in Supplementary Figure S4. Results indicated that the DEGs identified in leaves had the largest variation in expression. The up-regulated DEGs in roots are distributed in each stratum. However, the down-regulated DEGs in root fall into the 2–16 fold stratum. The majority of the DEGs in stems are distributed in either the down-regulated 2–16 fold or the up-regulated 2–8 fold stratum (Supplementary Figure S4). Interesting, expression of *PsnMYB108* responsive to salt stress was observed in both leaves and roots, with 35.2-fold up-regulation in leaves and 12.5-fold up-regulation in roots. So we selected this gene for further study.

### Spatiotemporal Expression Pattern of *PsnMYB108* Gene

**FIGURE 7:**
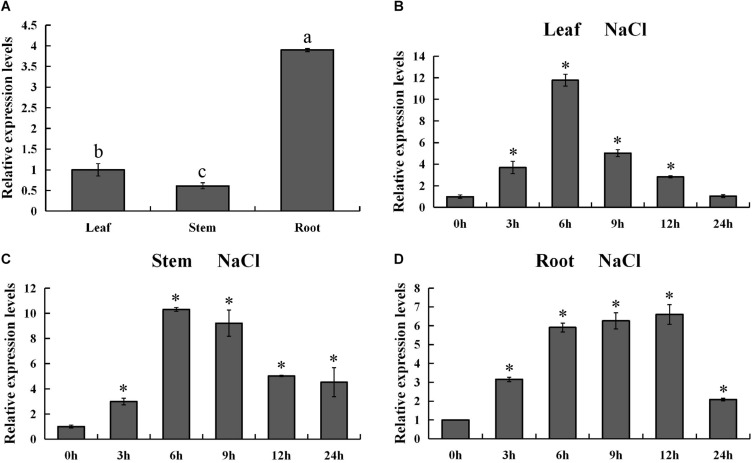
Analysis of spatiotemporal expression pattern of *PsnMYB108*. **(A)** Tissue-differential expression patterns of *PsnMYB108*. The expression level was calculated relative to its expression level in leaf. Three biological replicates were used. The error bars represent standard deviation. Statistical analysis was performed using one-way ANOVA, significant differences (*P* < 0.05) are indicated by different lower case letters. **(B–D)**, Expression analysis of *PsnMYB108* in response to salt stress in leaves, stems and roots. The expression level of each gene was calculated relative to its expression level at 0 h. The error bars represent standard deviation. The asterisk indicates significant differences between the treatment group and the control group (*t-*test, *P* < 0.05).

In order to validate the expression pattern of *PsnMYB108* gene across the tissues, we used qRT-PCR for gene expression quantification. The results showed that *PsnMYB108* was significantly differentially expressed in the root, stem and leaf tissues (Figure 7A). This is slightly different from the results of our RNA-Seq data mentioned above. The discrepancy might be caused by the different culture conditions of the experimental materials (hydroponics vs. soil culture). However, the expression trend of *PsnMYB108* in different tissues is consistent. For example, the expression level of *PsnMYB108* in roots is much higher than that in leaves and stems, and similar expression levels are observed between leaves and stems. In order to explore the spatiotemporal expression pattern of *PsnMYB108* in response to salt stress, we examined its expression in root, stem and leaf tissues after treatment with 150 mM NaCl for 0, 3, 6, 9, 12, and 24 h, respectively. As shown in Figures 7B–D, the expression of *PsnMYB108* showed a trend that gradually increases and then reverts, indicating that it is inducible by the salt stress with a peak at 6 or 12 h.

### Subcellular Localization of PsnMYB108

**FIGURE 8:**
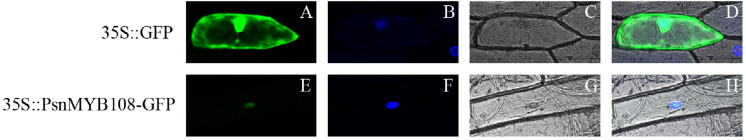
Subcellular localization analysis of PsnMYB108. We transferred the constructed fusion vector (35S:PsnMYB108-GFP) and control vector (35S:GFP) into onion epidermal cells by particle bombardment. **(A,E)** GFP fluorescence; **(B,F)** DAPI staining (nuclear staining); **(C,G)** bright fields; **(D,H)**, combined images of GFP fluorescence, DAPI staining and bright fields.

In order to validate subcellular localization of PsnMYB108, we performed particle bombardment experiment. Results indicated that the fluorescence signal of the 35S:GFP protein can be observed in the entire cell, while the fluorescence signal of the 35S:PsnMYB108-GFP protein can be observed only in the nucleus (Figure 8), demonstrating that the protein encoded by *PsnMYB108* gene is localized in the nuclear.

### Development and Stress Tolerance Analysis of *PsnMYB108* Transgenic Tobacco Plants

**FIGURE 9:**
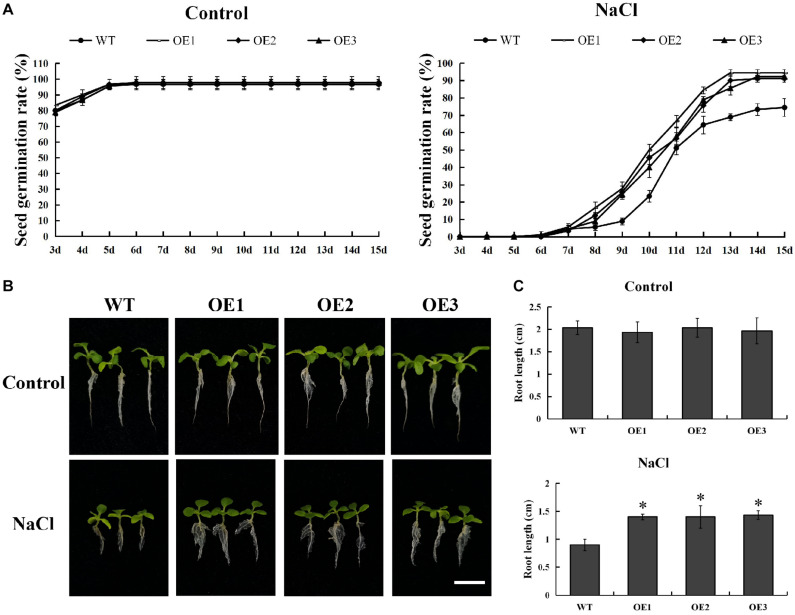
Comparison of seed germination rate and root length between wild-type lines and transgenic lines. **(A)** Seed germination rate of different lines under 0 or 150 mM NaCl. **(B)** Comparison of growth phenotypes among different lines under 0 or 150 mM NaCl. Scale bars = 1 cm. **(C)** Root length of different lines under 0 or 150 mM NaCl. Three biological replicates were used. The error bars represent standard deviation. Asterisks (*) indicate significant differences between transgenic lines and wild type lines (*t*-test, *P* < 0.05).

In order to explore its function, we successfully developed five transgenic tobacco plants overexpressing *PsnMYB108* by *Agrobacterium tumefaciens*-mediated transformation. The seeds of three randomly selected T3 generation transgenic lines (OE1-3) and wild-type tobacco were sown on medium containing 0 or 150 mM NaCl. The results showed that there was no significant difference in the germination rate between the wild-type and the transgenic plants on the medium without NaCl (Figure 9A). However, the germination rate of transgenic plants was significantly higher than that of the wild-type plants on the medium containing 150 mM NaCl (Figure 9A). After 15 days of cultivation, the seed germination rate of each line tended to be stable. The seed germination rate of the transgenic plants exceeded 90%, while that of the wild type plants was only 70–80%. In addition, the root length of transgenic plants was also significantly higher than that of the wild-type plants under 150 mM NaCl treatment (Figures 9B,C). These results indicated that overexpression of *PsnMYB108* can significantly improve the salt stress tolerance of tobacco plants.

### Analyze of Physiological Traits

**FIGURE 10:**
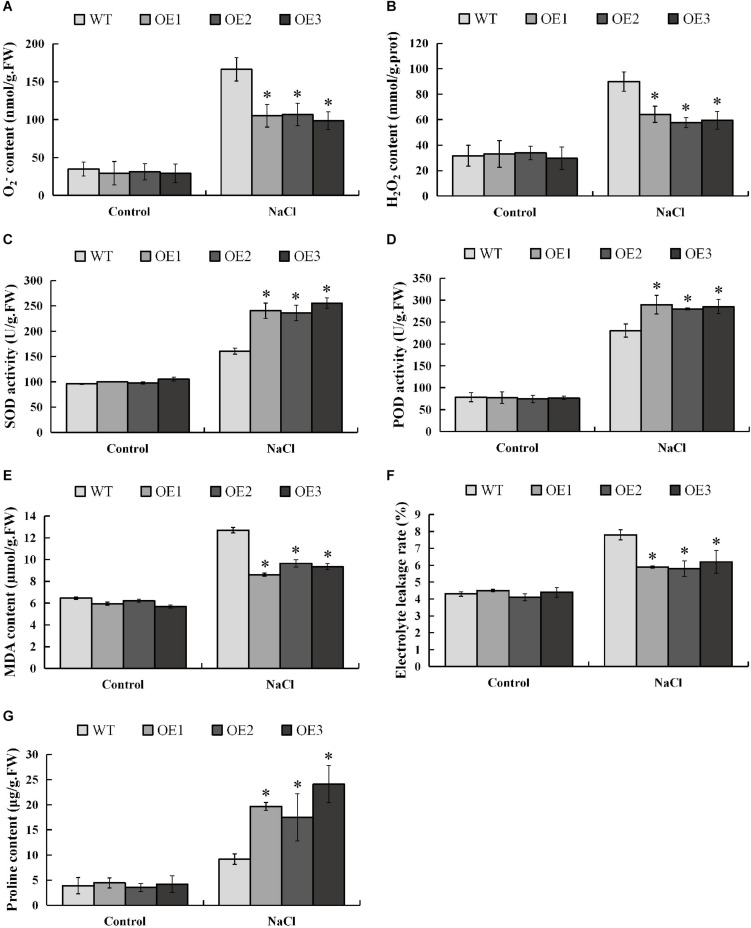
Measurement of physiological indexes. **(A–G)**, O_2_^–^ content, H_2_O_2_ content, SOD activity, POD activity, MDA content, electrolyte leakage rate, and proline content of different lines under different conditions. Three biological replicates were used. The error bars represent standard deviation. Asterisks (*) indicate significant differences between transgenic lines and wild type lines (*t*-test, *P* < 0.05).

When plants are subjected to salt stress, a large amount of reactive oxygen species (ROS) will be produced, and excessively accumulated ROS will be toxic to the plants. In order to investigate whether *PsnMYB108* will affect the plant’s ability to scavenge ROS under salt stress, we measured the contents of the two main ROS (O_2_^–^ and H_2_O_2_) in different plants before and after salt stress. The results showed that there was no significant difference in O_2_^–^ and H_2_O_2_ contents between transgenic and wild-type plants under normal condition, but the accumulations of O_2_^–^ and H_2_O_2_ in transgenic plants were significantly lower than those in wild-type plants under salt stress (Figures 10A,B). SOD and POD can remove O_2_^–^ and H_2_O_2_ in plants, respectively. So we measured the SOD and POD activities of transgenic and wild-type tobacco plants before and after salt stress. Results indicated that the SOD and POD activities of the transgenic and wild-type plants have no significant difference under normal condition (Figures 10C,D). However, the two traits of the transgenic plants are significantly higher than that of the wild-type plants under 150 mM NaCl. These results demonstrate that *PsnMYB108* can positively regulate the activities of SOD and POD under salt stress, reducing the accumulation of ROS in transgenic tobacco lines.

In addition, we evaluated the damage degree of cell membrane of the lines by salt stress, measuring the MDA (membrane lipid peroxidation product) content and electrolyte leakage rate before and after the stress (Figures 10E,F). The results indicated that under normal condition different lines displayed no significant difference in these two physiological traits. However, under 150 mM NaCl treatment, the traits are significantly lower in the transgenic tobacco lines than that in the wild-type plants. These lines of evidence demonstrated that under salt stress, the membrane damage degree in the transgenic lines is significantly lower than that of the wild type.

Salt causes plants to suffer osmotic stress, thereby affecting plant growth. Proline is an important osmotic regulator and also plays a role in the removal of ROS (Xu et al., 2018). Therefore, we determined the proline content in different strains before and after salt stress. The results showed that the proline content in the transgenic tobacco plants overexpressing the *PsnMYB108* gene was similar to that of the wild-type under normal condition; but the proline content in the transgenic tobacco plants was significantly higher than that in the wild-type plants under salt stress (Figure 10G). These results indicate that *PsnMYB108* may enhance the salt stress tolerance of plants by increasing the accumulation of proline in plants under salt stress.

### Expression of ABA-Responsive Genes

In order to explore whether overexpression of *PsnMYB108* gene will affect the expression of ABA-responsive genes in plants, we investigated the expression levels of *ABF2*, *RD29B*, *Rd22* in wild-type and transgenic tobacco plants with or without salt stress. The results showed that there was no significant difference in the expression levels of *ABF2*, *RD29B*, *Rd22* in wild-type and transgenic plants under the control condition (Supplementary Figure S5). However, the expression level of *ABF2* in transgenic plants was significantly higher than that in wild-type plants under salt stress, and the expression levels of *RD29B* and *Rd22* in transgenic plants were also higher than those in wild-type plants (Supplementary Figure S5).

## Discussion

The R2R3-MYB gene family widely exists in eukaryotes, and plays an important role in the regulation of primary and secondary metabolism, growth and development, hormone and stress response. In previous studies, 192 poplar R2R3-MYB proteins were identified from the Joint Genome Institute (JGI) Ptri version 1.1 database, based on the highly conserved DNA binding domain characteristics of the N-terminus of transcription factors (Wilkins et al., 2009). However, part of the Gene IDs used in the previous research were challenged to be found with the upgrade of the database, and the remaining members were unable to be mapped to the new Gene ID. With the improved genomic information, we can better identify new members of the family. In this study, we identified 207 R2R3-MYB genes in poplar, using the JGI Ptri version 3.0 (Tuskan et al., 2006; Goodstein et al., 2012). We found that the R2 and R3 MYB repeats of the proteins encoded by these genes share the same characteristics as the other species. In addition, there are many conserved sites in the C-terminal, which may function as the recognition and binding sites of target genes.

Based on phylogenetic analysis and previous research in Arabidopsis, we divided the poplar R2R3-MYB family into 23 groups. Chromosome distribution results show that these R2R3-MYB genes are unevenly distributed on 19 chromosomes. Previous studies have shown that poplar has experienced at least three rounds of whole genome duplication, followed by multiple segmental duplication, tandem duplication and transposition events (Tuskan et al., 2006; Chai et al., 2014). Therefore, we explored the tandem and segmental duplication events in poplar R2R3-MYB genes. The results indicated that 19 R2R3-MYB genes have 12 tandem duplication events on five chromosomes; 64 gene pairs have segmental duplication events, involving 126 genes distributed on 19 chromosomes. These results imply that tandem and segmental duplication events play an important role in the expansion of the R2R3-MYB gene family in poplar. In addition, we also explored the collinear relationships between the poplar R2R3-MYB genes and similar genes from Arabidopsis and rice. A total of 32 such poplar genes share orthologs with 27 Arabidopsis genes and with 2 rice genes. These conclusions provide insights into the evolutionary relationship of R2R3-MYB family genes.

Since many studies have shown that the R2R3-MYB transcription factors play significant roles in regulation of plant growth and development, as well as of abiotic stress responses, thus we explored their gene expression in response to salt stress and gene expression patterns across different tissues. Through tissue differential expression analysis, we identified eight genes that were differentially expressed in any two tissues of leaf, stem and root. We divided them into three groups with different expression patterns. Comparing the RNA-Seq data collected from both before and after salt stress, we identified that there were 65 DEGs in roots (with respective 36 and 29 genes up- and down-regulated), followed by 33 DEGs in leaves (with respective 17 and 16 genes), and by 15 DEGs in stems (with respective 7 and 8 genes) (Supplementary Data set S2). We then compared these DEGs across different tissues. We found that the majority of DEGs (47) were only differentially expressed in roots (Figure 5E), followed by 14 genes in leaves, and by only 3 genes in stems (Figure 5E). In addition to the seven genes that can respond to salt stress in all three tissues, there are nine genes responsive to salt stress in both leaves and roots, and three genes in both leaves and stems, and two genes in both stems and roots (Figure 5E).

Finally, we annotated these salt stress responsive genes to the Arabidopsis genome (Supplementary Table S4). The results indicated that many homologous genes in Arabidopsis have similar functions observed in poplar. For example, *AtDIV2*, a homologous gene of *Potri.010G240800.1* and *Potri.001G219100.1*, plays a negative role in salt stress and is required for ABA signaling in Arabidopsis (Fang et al., 2018). *AtMYB52* is a counterpart in Arabidopsis of *Potri.015G033600.1*. Overexpression of *AtMYB52* confers plant ABA hypersensitivity and drought resistance (Park et al., 2011). *AtMYB93*, the best matching gene for *Potri.005G074500.1* and *Potri.005G164900.1*, is involved in regulating the development of lateral roots in Arabidopsis (Gibbs and Coates, 2014).

In order to further verify the function of the genes we identified, we chose the *PsnMYB108* gene, which was significantly up-regulated in both roots and leaves under salt stress, for clone and genetic transformation. Evidence from qRT-PCR indicated that *PsnMYB108* was significantly differentially expressed across the tissues. In addition, evidence from time-course experiments under salt stress demonstrated that *PsnMYB108* gene expression displayed a consistent trend of up-regulation and then revert over tissues. Subsequently, we successfully cloned the gene and developed transgenic tobacco plants overexpressing *PsnMYB108*. Stress tolerance experiments confirmed that overexpression of *PsnMYB108* in tobacco can significantly improve salt stress tolerance.

When plants are subjected to abiotic stresses, excess ROS accumulated in the body will have a toxic effect on itself, so the ability of ROS scavenging is very important for plants to cope with abiotic stresses. Therefore, we measured the O_2_^–^ content, H_2_O_2_ content, SOD activity and POD activity of transgenic and wild-type tobacco plants under salt stress. Without the stress, the physiological traits of both transgenic and wild-type plants showed no significant difference. Under salt stress, however, the O_2_^–^ and H_2_O_2_ contents of the transgenic plants were significantly lower than that of the wild-type plants; the SOD and POD activities of the transgenic plants were significantly higher than that of the wild-type plants. These results indicate that *PsnMYB108* may enhance tolerance in the transgenic plants to salt stress by increasing SOD and POD activities, in order to reducing excess accumulated ROS.

Malondialdehyde as a product of membrane lipid peroxidation can indirectly reflect the damage degree of cell membrane (Tsikas, 2017). The electrolyte leakage rate can also assess cell membrane damage. In order to further evaluate the stress resistance of *PsnMYB108* overexpressed plants, we measured the MDA content and electrolyte leakage rate of both the transgenic tobacco and wild-type plants. As a result, both the transgenic and wild-type plants displayed no significant difference in these two traits without salt stress. However, under salt stress the two traits in the transgenic plants were significantly lower than that in the wild-type plants, further indicating that cell damage of the transformants was significantly lower than that of the wild-type. These lines of evidence demonstrate that *PsnMYB108* overexpressing tobacco lines have stronger salt stress tolerance than the wild-type plants.

Proline plays an important role in stabilizing the structure of biological macromolecules, maintaining the stability of biological membranes, regulating osmotic balance, and removing ROS (Natarajan et al., 2012). Many plants accumulate large amounts of proline to improve tolerance when exposed to salt stress (Watanabe et al., 2000). Our experimental results show that the proline content in transgenic tobacco plants overexpressing *PsnMYB108* is significantly higher than that in wild-type plants under salt stress. This implies that *PsnMYB108* may regulate the process of osmotic balance by increasing the accumulation of proline in plants under salt stress, thereby improving the salt tolerance of plants.

ABA is widely involved in the complex regulatory network regulating plant stress response and development (León et al., 2014). ABA signal transduction pathway is the core of plant response to drought and salt stresses (Zhu, 2002). When plants are subjected to abiotic stresses, they can induce the accumulation of ABA to enhance stress tolerance (Xiong et al., 2002; Zhu, 2002). Previous studies have shown that some ABA-responsive genes can participate in the ABA signal transduction pathway to enhance the abiotic stress tolerance of plants (Uno et al., 2000). In this study, we found that the expression levels of ABA-responsive genes *RD29B*, *Rd22* in transgenic tobacco plants overexpressing *PsnMYB108* were higher than those in wild-type plants under salt stress condition, and the expression level of *ABF2* was significantly higher than that in wild-type plants. In addition, studies have shown that H_2_O_2_ can interact with many signal molecules and play an important role in various stress responses (Saxena et al., 2016). In future research, we will transfer *PsnMYB108* into poplar to explore whether it is involved in the ABA signal transduction pathway or modulates H_2_O_2_ signaling molecules to improve plant salt stress tolerance.

The results of phylogenetic analysis showed that PsnMYB108 belongs to the S20 group. Interestingly, we found many genes in this group that play an important role in response to abiotic stress. For example, the Arabidopsis *bos1* mutant is sensitive to salt, drought and oxidative stresses (Mengiste et al., 2003). The Arabidopsis transcription factor MYB112 promotes anthocyanin formation during salinity and under high light stress, and anthocyanin is an antioxidant that scavenges ROS (Lotkowska et al., 2015). *AtMYB2* gene is induced by drought and ABA treatment, and AtMYB2 protein acts as a transcriptional activator in ABA signaling (Abe et al., 2003). Since genes with close genetic relationships may have similar functions, we speculate that *PsnMYB108* may respond to multiple abiotic stresses and play a role in promoting anthocyanin formation or ABA signal transduction pathway. But this requires us to further verify in poplar.

## Conclusion

In this study, we focused on 207 poplar R2R3-MYB family members, starting with conserved domain characterization, phylogenetic analysis, chromosome distribution and collinearity analysis, and then we explored the tissue differential expression patterns and salt stress response of these genes, using RNA-Seq data. Among these genes, we found the *PsnMYB108* gene that was significantly up-regulated in both root and leaf tissues under salt stress. Subsequently, we cloned this gene and successfully transferred it into tobacco. We validated that the gene displayed a tissue differential expression pattern, and showed a trend of up-regulation followed by reversion in response to salt stress. Evidence from both morphological evaluation and physiological experiments indicated that the transformants can significantly improve salt stress tolerance by increasing the scavenging capacity of ROS and the accumulation of proline. Interestingly, through phylogenetic analysis, we found that the genes of the same group of *PsnMYB108* can play an important role in responding to abiotic stresses by promoting the formation of anthocyanins or participating in the ABA signal transduction pathway. In the future, we will further explore the molecular mechanism of *PsnMYB108* in response to salt stress in these aspects. Collectively, our research laid the foundation for future functional studies of the poplar R2R3-MYB gene family.

## Data Availability Statement

All datasets presented in this study are included in the article/Supplementary Material.

## Author Contributions

TJ and BZ designed research. KZ conducted experiments, data analysis, and wrote the manuscript. ZC, QG, WY, and HL conducted data analysis. All authors read and approved the manuscript.

## Conflict of Interest

The authors declare that the research was conducted in the absence of any commercial or financial relationships that could be construed as a potential conflict of interest.

## Supplementary Material

Supplementary Figure 1 The DNA binding domain alignment of poplar R2R3-MYB family.

Supplementary Figure 2 The DNA binding domain alignment logo of poplar R2R3-MYB family. **(A)**, logo of R2 repeat; **(B)** logo of R3 repeat.

Supplementary Figure 3 NJ-phylogenetic tree with bootstrap values.

Supplementary Figure 4 Barplot of the DEGs in response to salt stress by fold changes.

Supplementary Figure 5 The expression levels of *ABF2*, *RD29B*, *Rd22* of different lines with or without NaCl treatments. The expression level of each gene was calculated relative to its expression level in WT. Three biological replicates were used. The error bars represent standard deviation. Asterisks indicate significant differences between transgenic lines and wild type lines (*t*-test, *P* < 0.05).

Supplementary Table 1 Primer sequences used in this study.

Supplementary Table 2 Tandemly and segmentally duplicated poplar R2R3-MYB gene pairs.

Supplementary Table 3 List of syntenic gene pairs.

Supplementary Table 4 Annotations of differentially expressed genes.

Supplementary Data set 1 Pairwise gene expressions. Leaf (C), leaves without treatment; Stem (C), stems without treatment; Root (C), roots without treatment. The DEGs were identified using DESeq software and the thresholds were fold change ≥ 2 and padj (*p*-value adjusted for multiple testing) ≤ 0.05.

Supplementary Data set 2 DEGs in response to salt stress. Leaf (C), leaves without treatment; Stem (C), stems without treatment; Root (C), roots without treatment; Leaf (T), leaves with salt treatment; Stem (T), stems with salt treatment; Root (T), roots with salt treatment. DEGs were identified using DESeq software and the thresholds were fold change ≥ 2 and padj (*p*-value adjusted for multiple testing) ≤ 0.05.
