# Supplementary material for: Characterization of the Poplar R2R3-MYB Gene Family and Over-Expression of PsnMYB108 Confers Salt Tolerance in Transgenic Tobacco
Source: Front Plant Sci. 2020 Oct 16;11:571881. doi: 10.3389/fpls.2020.571881 (PMC7596293; doi:10.3389/fpls.2020.571881)

R2 DNA binding domain alignment

```
                           10         20         30         40         50         60
                  ....|....| ....|....| ....|....| ....|....| ....|....| ....|....| ....|....
Potri001G005100.1 RGPWTPEEDE LLVNYIKKE- ---GEG-RWR T-----LPKK ---------A GLLRCGKSCR LRWMNYL--
Potri001G035800.1 -KPWSREEIE LLRKGIQKY- -PKGTSRRWE V-----ISEY IGT------- -----GRSVE EI-------
Potri001G036000.1 KGPWTSAEDA ILIEYVKKH- ---GEG-NWN A-----VQKH ---------S GLFRCGKSCR LRWANHL--
Potri001G075400.1 RGLWSPEEDE KLIKYITIH- ---GHG-SWS S-----VPNL ---------A GLERCGKSCR LRWINYL--
Potri001G086700.1 RGPWTTKEDT LLINYIQAH- ---GEG-HWR S-----LPKK ---------A GLLRCGKSCR LRWMNYL--
Potri001G099800.1 RGLWSPEEDE KLIRYITTH- ---GYG-CWS E-----VPEK ---------A GLQRCGKSCR LRWINYL--
Potri001G118800.1 KGPWTAEEDK KLINFILTN- ---GQC-CWR A-----VPKL ---------A GLRRCGKSCR LRWTNYL--
Potri001G139900.1 KGPWTPEEDE KLVDYIKRN- ---GHE-NWK A-----LPKL ---------A GLNRCGKSCR LRWTNYL--
Potri001G169600.1 KGAWTAEEDR KLAEVIATH- ---GAK-RWR T-----IASK ---------A ALNRCGKSCR LRWLNYL--
Potri001G197000.1 RGLWSPEEDE KLANYISTY- ---GHG-CWS S-----VPKL ---------A GLQRCGKSCR LRWINYL--
Potri001G219100.1 -SSWSRLEDK QFEQALVLF- -PEETPRRWE K-----ISSY VP-------- -----GKSWR EVRKHYED-
Potri001G224500.1 KGPWTSAEDA ILIDYVKKH- ---GEG-NWN S-----VQKH ---------S GLFRCGKSCR LRWANHL--
Potri001G235500.1 KGPWTEKEDI LLINFVHLF- ---GDR-RWD S-----IAKV ---------S GLNRTGKSCR LRWVNYL--
Potri001G248800.1 --KWTPAENK AFENALAVY- -DEDTPDRWH K-----VAAM IP-------- -----GKTVG DVIKQYK--
Potri001G250000.1 KGQWTEEEDR KLIRLVKQF- ---GVR-KWA QI----AEKL D--------- --GRAGKQCR ERWHNHL--
Potri001G258700.1 KGLWSPEEDD KLMNYILNN- ---GQG-CWS D-----VARN ---------A GLQRCGKSCR LRWINYL--
Potri001G267300.1 KGLWSPEEDE KLIKYMLTN- ---GQG-CWS E-----IARN ---------A GLQRCGKSCR LRWINYL--
Potri001G300200.1 KGPWSPEEDA LLKHLVIKH- ---GPR-SWT MI----ARAV P--------- --GRSGKSCR LRWCNQ---
Potri001G336700.1 RGPWTIEEDH KLTNFILNN- ---GIQ-CWR M-----VPKL ---------A GLLRCGKSCR LRWINYL--
Potri001G346600.1 -GCWTLEEDL ILTNYIANH- ---GEG-VWN S-----LAKA ARLQKLQV-I CLKRTGKSCR LRWLNYL--
Potri001G347200.1 KGQWTPQEDR LLVQSVKQY- ---GIK-KWS QI----AKML E--------- --GRVGKQCR ERWHNHL--
Potri001G408700.1 KGPWTEEEDK KLISYIQKH- ---GHG-RWR L-----LPKN ---------A GLKRCGKSCR LRWTNYL--
Potri001G470500.1 RGQWTPEEDN KLSSYIAQH- ---GTR-NWR L-----IPKN ---------A GLQRCGKSCR LRWTNYL--
Potri002G038500.1 RGPWTAEEDR ILINHIQLY- ---GHG-NWR A-----LPKQ ---------A GLLRCGKSCR LRWINYL--
Potri002G073500.1 RGHWRPAEDD KLRQLVEQY- ---GAQ-NWN DI----AEKL Q--------- --GRSGKSCR LRWFNQ---
Potri002G096800.1 KGPWTPEEDE KLVDHIKKH- ---GQG-SWR A-----LPKL ---------A GLNRCGKSCR LRWTNYL--
Potri002G113700.1 KGPWSPEEDA KLKSYIEQH- ---GTGGNWI A-----LPQK IDIGVVSE-S GLKRCGKSCR LRWLNYL--
Potri002G122600.1 KGPWSPEEDE ALQRLVQTY- ---GPR-NWS LI----SKSI P--------- --GRSGKSCR LRWCNQ---
Potri002G128900.1 KGPWSSEEDM ILTGLVERH- ---GPK-NWS LI----SRYI K--------- --GRSGKSCR LRWCNQ---
Potri002G140900.1 KGPWKAEEDE VLIMHVKKY- ---GPR-DWS SIR---SKGL L--------- --QRTGKSCR LRWVN-----
Potri002G157600.1 KGPWTPEEDD LLVTYIKKN- ---GHG-SWR S-----LPKL ---------A GLLRCGKSCR LRWTNYL--
Potri002G173900.1 RGPWTPREDA LLTEYIQAH- ---GEG-HWR S-----LPKK ---------S GLLRCGKSCR LRWMNYL--
Potri002G185900.1 KGLWSPEEDE KLLSHITKY- ---GHG-CWS S-----VPKQ ---------A GLQRCGKSCR LRWINYL--
Potri002G191800.1 KGPWTIEEDS LLAHYITIH- ---GEG-HWN S-----AARC AG-------- -LKRTGKSCR LRWLNYL--
Potri002G198100.1 KGRWTAEEDE KLAKYIQAN- ---GEG-SWR S-----MPKN ---------A GLLRCGKSCR LRWINYL--
Potri002G228700.1 KGPWTVAEDA ILIEYVKKH- ---GEG-NWN S-----VQKN ---------F RLMRCGKSCR LRWANHL--
Potri003G064600.1 KGAWTAEEDR KLAEVIAIH- ---GAR-KWK T-----IAAK ---------A ALNRCGKSCR LRWLNYL--
Potri003G079100.1 KGAWTAEEDN KLAHCVEVH- ---GAK-RWK T-----VALK ---------S GLNRCGKSCR LRWMNYL--
Potri003G094200.1 KGPWTPDEDE KLIDYIKRN- ---GHE-NWK A-----LPML ---------A GLNRCGKSCR LRWTNYL--
Potri003G114100.1 KGPWTAEEDK KLINFILTN- ---GQC-CWR A-----VPKL ---------A GLRRCGKSCR LRWTNYL--
Potri003G123800.1 KGTWTKEEDD CIMELVGKH- ---GCR-KWS VI----AKSL P--------- --GRVGKQCR ERWFNHL--
Potri003G132000.1 RGLWSPEEDE KLIRYITTH- ---GYG-CWS E-----VPEK ---------A GLQRCGKSCR LRWINYL--
Potri003G144200.1 RGPWTTREDT LLINYIQAH- ---GEG-HWR S-----LPKK ---------A GLLRCGKSCR LRWMNYL--
Potri003G144300.1 RGPWTTREDA LLVNYIQKH- ---GEG-HWR S-----LPNK ---------A GLLRCGKSCR LRWLNYL--
Potri003G155700.1 RGLWSPDEDE KLIKYITTH- ---GHG-SWS S-----VPKL ---------A GLQRCGKSCR LRWINYL--
Potri003G168900.1 -SSWTPEEYD LIRKFHEKH- ---GSD--WK T-----LAEA LG-------- ---KHRFHVK DTWRR----
Potri003G189700.1 KGPWTSAEDA ILIEYVKKH- ---GEG-NWN S-----VQKH ---------S GLFRCGKSCR LRWANHL--
Potri003G219900.1 RGPWTPEEDE LLANYIKKE- ---GEG-RWR T-----LPKK ---------A GLLRCGKSCR LRWMNYL--
Potri004G026600.1 RGAWSPEEDQ KLIAYINRH- ---GIR-NWI E-----MPKA ---------A GLLRSGKSCR LRWMNYL--
Potri004G033100.1 KGPWTPEEDQ KLVDYIQKH- ---GYG-NWR T-----LPKN ---------A GLQRCGKSCR LRWTNYL--
Potri004G086300.1 KGPWTSDEDK KLINFILAN- ---GQC-CWR A-----VPKL ---------A GLLRCGKSCR LRWTNYL--
Potri004G088100.1 KGAWSTQEDQ KLIDYIQTH- ---GEG-CWR S-----LPEA ---------A GLHRCGKSCR LRWINYL--
Potri004G102600.1 RQRWQPEEDA VLRAYVKQY- ---GPK-EWN L-----ISQR VEATG----K TLNRDPKSCL ERWKNYL--
Potri004G115600.1 KGPWTPEEDK LLSEYVSSN- ---GEG-RWS S-----VSRC ---------S GLNRSGKSCR LRWVNYL--
Potri004G118000.1 KGPWTPEEDK LLSEYVSLN- ---GEG-RWS S-----VSRC ---------S GLNRSGKSCR LRWVNYL--
Potri004G126700.1 KGPWTPEEDI ILVSYIQEH- ---GPG-NWR A-----VPTN ---------T GLLRCSKSCR LRWTNYL--
Potri004G138000.1 KGAWTKEEDE RLVNYIKAQ- ---GEG-CWR S-----LPKA ---------A GLLRCGKSCR LRWINYL--
Potri004G174400.1 KGAWTKEEDD RLIAYIRTH- ---GEG-CWR S-----LPKA ---------A GLLRCGKSCR LRWINYL--
Potri004G215100.1 KGPWSPEEDS KLKEYIEKY- ---GTGGNWI A-----LPQK A--------- GLKRCGKSCR LRWLNYL--
Potri005G001600.1 KGLWSPEEDE KLLNYITKH- ---GHG-CWS S-----VPKQ ---------A DLQRCGKSCR LRWINYL--
Potri005G063200.1 RGHWRPAEDE KLRQLVDQY- ---GPH-NWN FI----AEHL Q--------- --GRSGKSCR LRWYNQL--
Potri005G074500.1 KGPWTPEEDQ KLVKYIQKH- ---GHG-SWR A-----LPKL ---------A GLNRCGKSCR LRWTNYL--
Potri005G087700.1 ---WTKEQDK AFENALATY- -PEDTSDWWE K-----ITAD VP-------- -----GKTVE EIKHHY---
Potri005G096600.1 RGPWSPAEDL RLITFIQKH- ---GHE-NWR A-----LPKQ ---------A GLLRCGKSCR LRWINYL--
Potri005G112000.1 KGAWTREEDK RLVAYIQAH- ---GEG-CWR S-----LPKS ---------A GLLRCGKSCR LRWINYL--
Potri005G118500.1 KGQWTAEEDS LLIRLVDEF- ---GIR-KWS HI----AQIF P--------- --GRIGKQCR ERWHNHL--
Potri005G142600.1 KGPWSPEEDE ALKKLVQRH- ---GPR-NWS LI----SKSI P--------- --GRSGKSCR LRWCNQ---
Potri005G164900.1 KGPWTPEEDQ KLVDYIQKH- ---GHG-SWR A-----LPKL ---------A DLNRCGKSCR LRWTNYL--
Potri005G186400.1 RGHWRPAEDD KLRQLVEQY- ---GAQ-NWN SI----AEKL Q--------- --GRSGKSCR LRWFNQ---
Potri005G224100.1 KGPWTAEEDQ ILINYIQLH- ---GHG-NWR A-----LPKQ ---------A GLLRCGKSCR LRWINYL--
Potri006G066400.1 KGLWKPEEDL ILKTYVETH- ---GEG-NWS T-----VSKR ---------S GLMRCGKSCR LRWKNYL--
Potri006G085900.1 RQRWRAEEDA LLRAYVKQY- ---GPR-EWN L-----VSQR MNTP------ -LNRDAKSCL ERWKNYL--
Potri006G097300.1 --KWTPEENK QFENALALY- -DKDTPDRWL K-----VAAL IP-------- -----GKTVD DVIKQYRE-
Potri006G122100.1 KGSWQEEEDE RLTASATLL- ---GER-KWD S-----IARL ---------S GLMRSGKSCR MRWLNYL--
Potri006G123400.1 KGPWSPEEDA KLKEYLEKQ- ---GTGGNWI A-----LPQK A--------- GLKRCGKSCR LRWLNYL--
Potri006G170800.1 RGPWSPEEDA KLKAYIDHF- ---GTGGNWI A-----LPQK I--------- GLKRCGKSCR LRWLNYL--
Potri006G221200.1 RGAWTAEEDQ KLAQVIEIH- ---GPK-RWR S-----VAAK ---------A GLNRCGKSCR LRWMNHL--
Potri006G221500.1 RGTWTATEDK ILTAYVRNY- ---GEG-NWA R-----VPKE ---------T GLKRCGKSCR LRWLNYL--
Potri006G221800.1 RGAWTALEDK ILTAYIKAH- ---GEG-KWR N-----LPKR ---------A GLKRCGKSCR LRWLNYL--
Potri006G234200.1 RGPWSPEEDA TLKSYLETH- ---GTGGNWI A-----LPQK A--------- GLKRCGKSCR LRWLNYL--
Potri006G275900.1 KGAWTPLEDE MLVDYVKIH- ---GEG-KWS N-----IVKE ---------T GLKRCGKSCR LRWMNYL--
Potri007G007900.1 KGPWSPEEDA ILKAYIEQH- ---GTGGNWI A-----LPQK I--------- GLKRCGKSCR LRWLNYL--
Potri007G048900.1 KGPWSPEEDE ALQKLVQKH- ---GAR-NWS LI----SKSI P--------- --GRSGKSCR LRWCNQ---
Potri007G064600.1 RVKWTLEENK LFENAIAEF- -DPGSPDFFE K-----ISER IP-------- -----EKTLK QTEDHFL--
Potri007G067600.1 RGPWSPAEDL RLIAFIQKH- ---GHE-NWR A-----LPKQ ---------A GLLRCGKSCR LRWINYL--
Potri007G076200.1 ---WTREHDK AFENALATY- -PEDASDRWE K-----IAED VP-------- -----GKTLE EIKHHY---
```

R3 DNA binding domain alignment

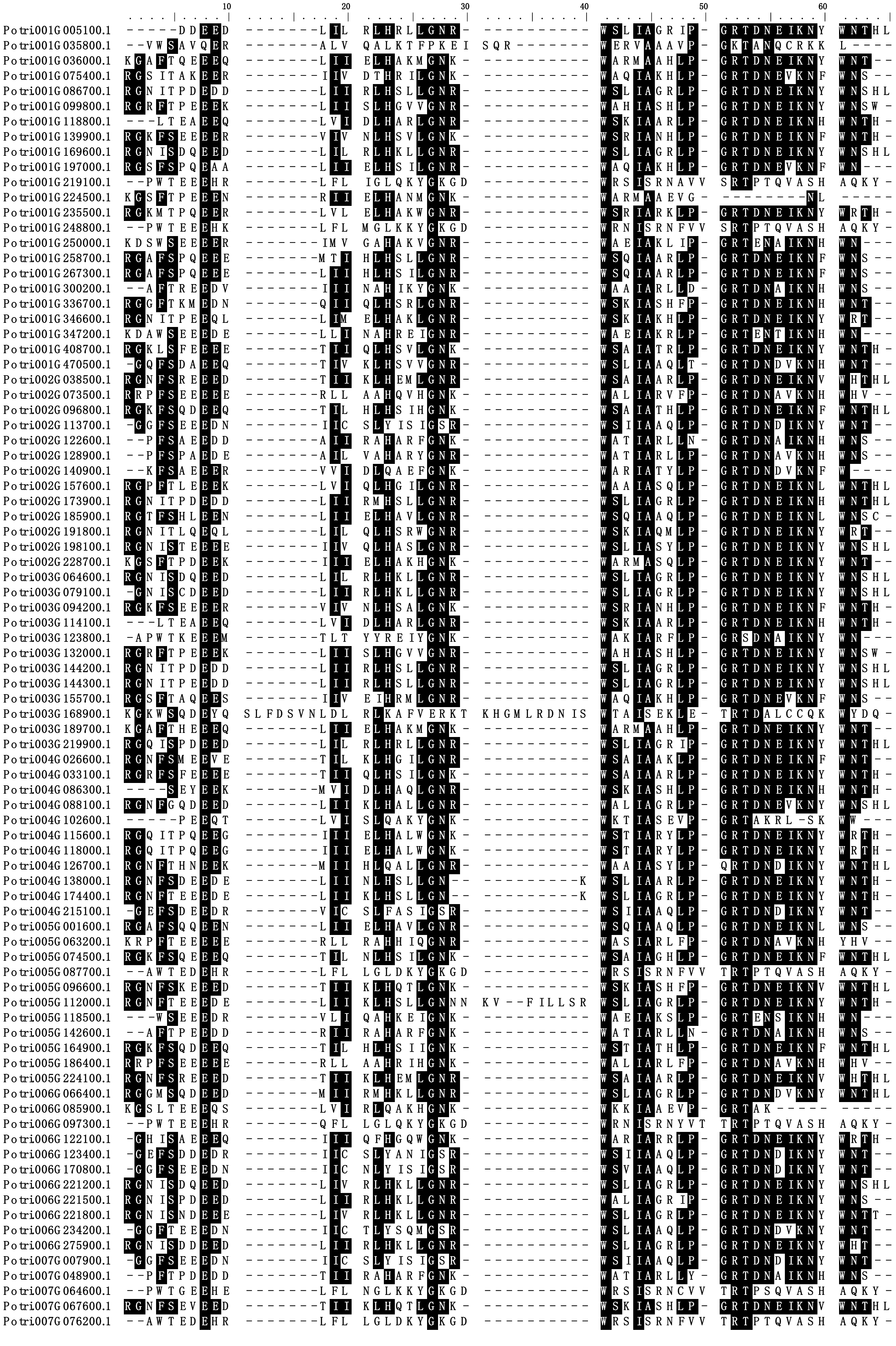

Supplement: Supplementary Figure 1 — The DNA binding domain alignment of poplar R2R3-MYB family. [file Image_1.PDF]
