# Supplementary figures and images for: Characterization of the Poplar R2R3-MYB Gene Family and Over-Expression of PsnMYB108 Confers Salt Tolerance in Transgenic Tobacco
Source: Front Plant Sci. 2020 Oct 16;11:571881. doi: 10.3389/fpls.2020.571881 (PMC7596293; doi:10.3389/fpls.2020.571881)

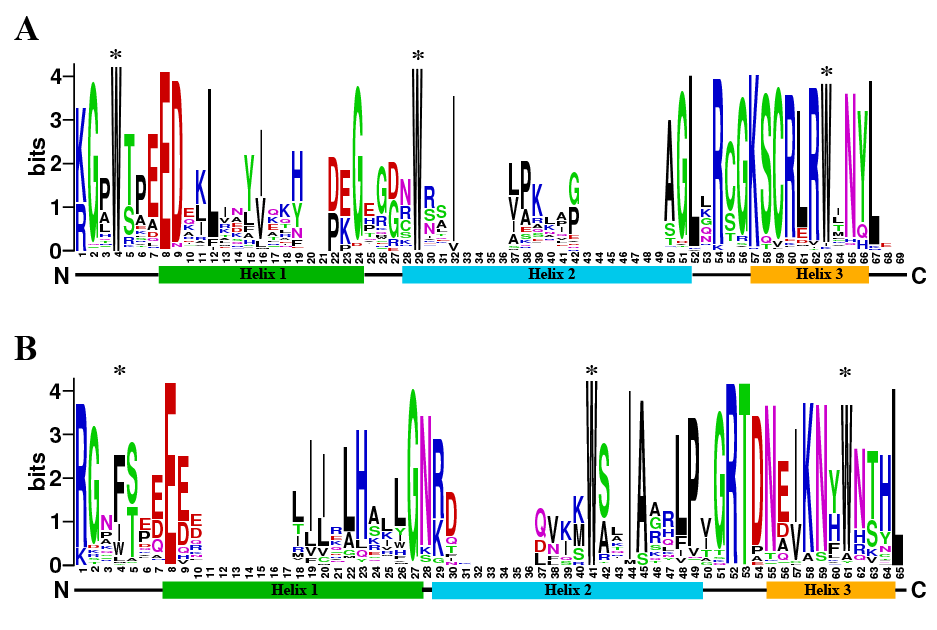

Supplement: Supplementary Figure 2 — The DNA binding domain alignment logo of poplar R2R3-MYB family. (A), logo of R2 repeat; (B) logo of R3 repeat. [file Image_2.PNG]

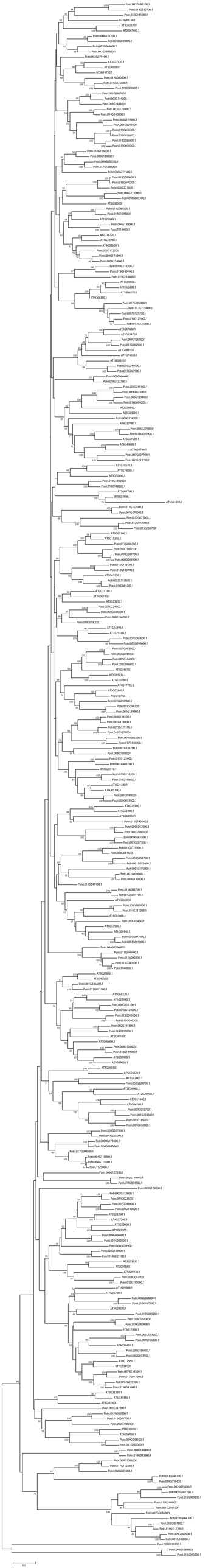

Supplement: Supplementary Figure 3 — NJ-phylogenetic tree with bootstrap values. [file Image_3.PDF]

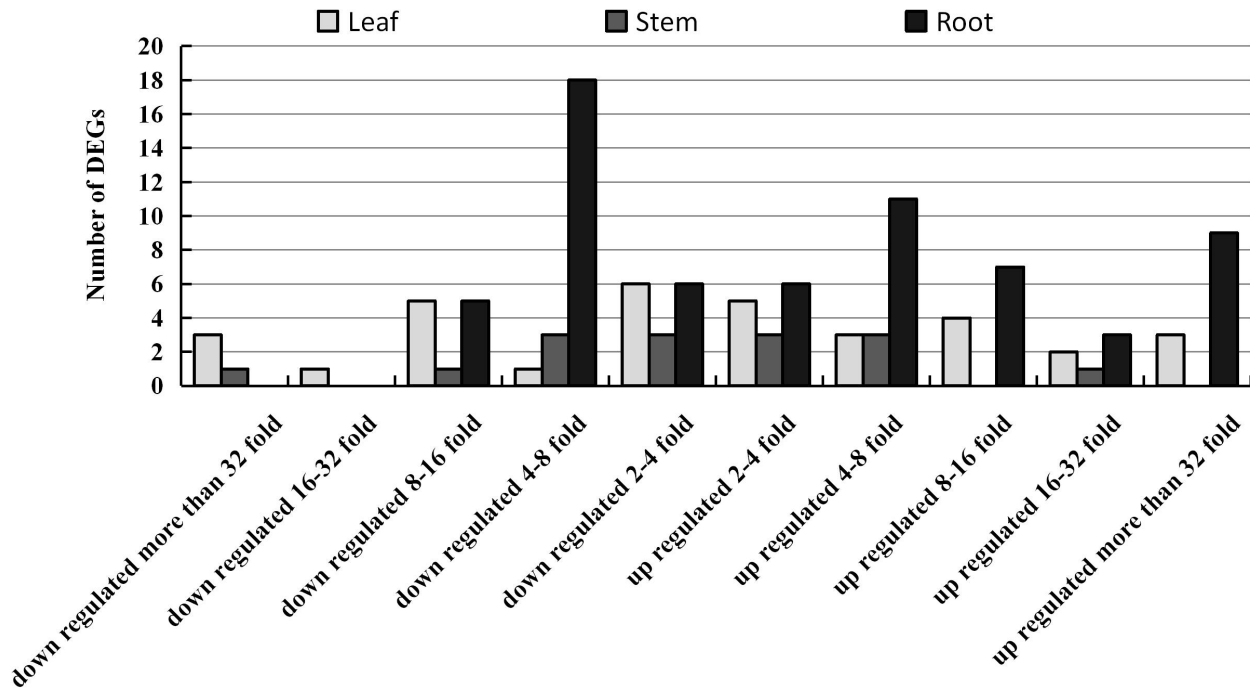

Supplement: Supplementary Figure 4 — Barplot of the DEGs in response to salt stress by fold changes. [file Image_4.PDF]

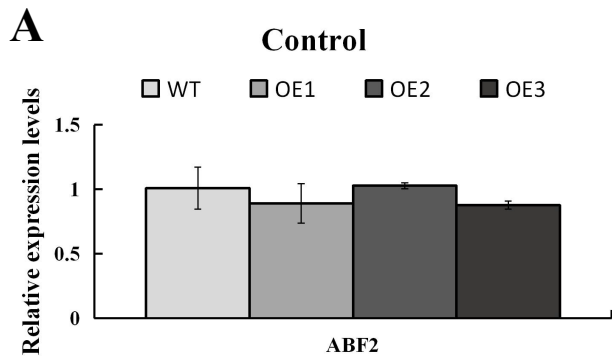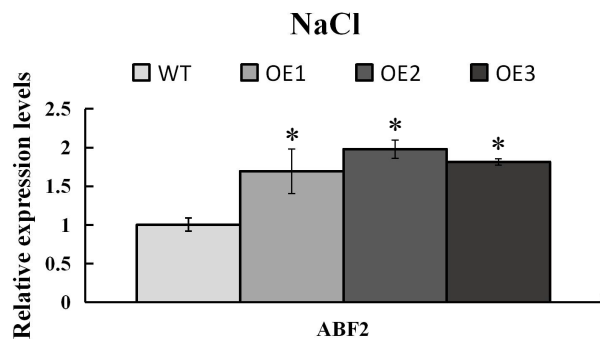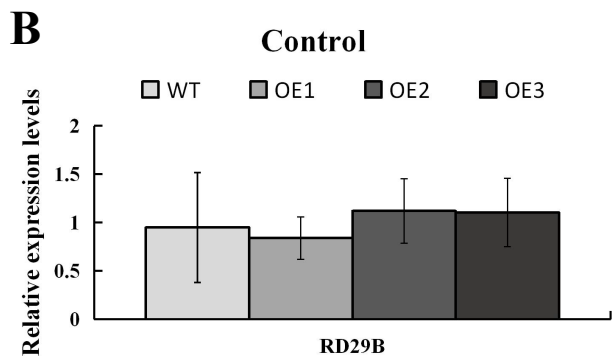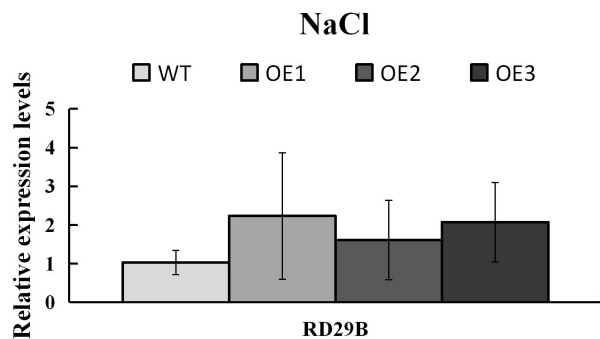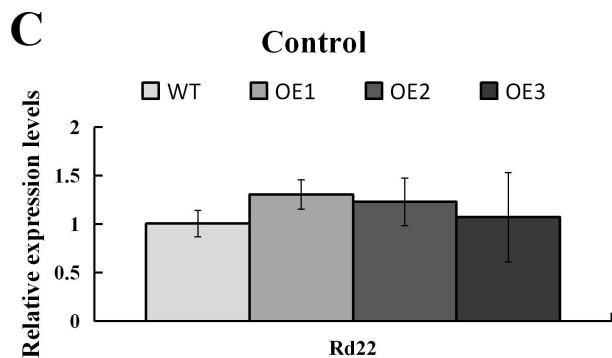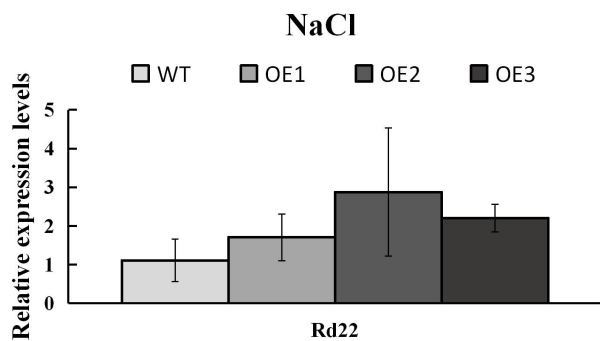

Supplement: Supplementary Figure 5 — The expression levels of ABF2, RD29B, Rd22 of different lines with or without NaCl treatments. The expression level of each gene was calculated relative to its expression level in WT. Three biological replicates were used. The error bars represent standard deviation. Asterisks indicate significant differences between transgenic lines and wild type lines (t-test, P < 0.05). [file Image_5.PDF]
