# Supplementary material for: Characterization of the Poplar R2R3-MYB Gene Family and Over-Expression of PsnMYB108 Confers Salt Tolerance in Transgenic Tobacco
Source: Front Plant Sci. 2020 Oct 16;11:571881. doi: 10.3389/fpls.2020.571881 (PMC7596293; doi:10.3389/fpls.2020.571881)
Supplement: Supplementary Table 1 — Primer sequences used in this study. [file Table_1.DOC]

Primer sequences for gene cloning

| ID | Name | 5’ primers | 3’ primers |
| --- | --- | --- | --- |
| Potri.010G149900 | *PsnMYB108* | ATGATGGATGTTGAAGGCAACAGC | TCACATGTTGTAATTCATCTGCTGCTG |

Primer sequences for vector construction

| Vector | 5’ primers | 3’ primers |
| --- | --- | --- |
| pROKII-PsnMYB108(Overexpression vector) | GCTCTAGACAAGTGGTTCTCCGATTAAG | CGAGCTCCCTTCACTCTCACCCATCTTACATC |
| pBI121-PsnMYB108-GFP | GCTCTAGAATGATGGATGTTGAAGGCAACAGC | GACTAGTCATGTTGTAATTCATCTGCTGCTG |

Primer sequences for qRT-PCR

| Name | 5’ primers | 3’ primers |
| --- | --- | --- |
| *ACT* | ACCCTCCAATCCAGACACTG | TTGCTGACCGTATGAGCAAG |
| *PsnMYB108*  *TUB*  *Rd22*  *RD29B*  *ABF2* | GGACTCTGAGTAACCACCTTGGTC  ATGAGAGAGTGCATATCGAT  GACTGTCCAAGAATGTGAAGAG  CAGGGTCACACGATCAATTTGC  GGAATGCTGAGGAAAGTCAAAC | GGTGTCTCACATGTTACACCAAG  TTCACTGAAGAAGGTGTTGAA  GACACTGGTTGCACTTCGTTTC  GTCCTGAGGTGAATCTTCTGGC  GGTAAAGTCAAAGAGCCTTGTC |
