# Supplementary material for: Characterization of the Poplar R2R3-MYB Gene Family and Over-Expression of PsnMYB108 Confers Salt Tolerance in Transgenic Tobacco
Source: Front Plant Sci. 2020 Oct 16;11:571881. doi: 10.3389/fpls.2020.571881 (PMC7596293; doi:10.3389/fpls.2020.571881)
Supplement: Supplementary Table 2 — Tandemly and segmentally duplicated poplar R2R3-MYB gene pairs. [file Table_2.DOC]

Tandemly and segmentally duplicated poplar R2R3-MYB gene pairs

| Gene ID | Gene ID | Duplication Type | Ka | Ks | Ka/Ks | Selection pressure |
| --- | --- | --- | --- | --- | --- | --- |
| Potri.003G144200.1  Potri.011G040200.1  Potri.011G040300.1  Potri.013G056400.1  Potri.013G149100.1  Potri.017G125600.1  Potri.017G125700.1  Potri.017G125800.1  Potri.017G125900.1  Potri.019G036300.1  Potri.019G118700.1  Potri.019G118800.1   | Potri.001G086700.1 | | --- | | Potri.001G075400.1 | | Potri.001G086700.1 | | Potri.001G099800.1 | | Potri.001G118800.1 | | Potri.001G139900.1 | | Potri.001G169600.1 | | Potri.001G005100.1 | | Potri.001G036000.1 | | Potri.001G267300.1 | | Potri.001G300200.1 | | Potri.001G235500.1 | | Potri.001G248800.1 | | Potri.001G250000.1 | | Potri.001G258700.1 | | Potri.001G470500.1 | | Potri.002G038500.1 | | Potri.002G073500.1 | | Potri.002G096800.1 | | Potri.002G122600.1 | | Potri.002G128900.1 | | Potri.002G140900.1 | | Potri.002G157600.1 | | Potri.002G173900.1 | | Potri.002G185900.1 | | Potri.002G191800.1 | | Potri.002G198100.1 | | Potri.004G215100.1 | | Potri.004G174400.1 | | Potri.004G086300.1 | | Potri.004G088100.1 | | Potri.004G102600.1 | | Potri.004G026600.1 | | Potri.004G033100.1 | | Potri.005G142600.1 | | Potri.005G063200.1 | | Potri.005G074500.1 | | Potri.005G087700.1 | | Potri.005G096600.1 | | Potri.005G001600.1 | | Potri.006G066400.1 | | Potri.006G170800.1 | | Potri.006G097300.1 | | Potri.007G134500.1 | | Potri.008G122100.1 | | Potri.008G128500.1 | | Potri.008G148400.1 | | Potri.008G173400.1 | | Potri.008G062700.1 | | Potri.008G081600.1 | | Potri.008G088000.1 | | Potri.008G089700.1 | | Potri.008G101400.1 | | Potri.012G055600.1 | | Potri.012G127700.1 | | Potri.012G140700.1 | | Potri.012G072500.1 | | Potri.012G080400.1 | | Potri.012G082000.1 | | Potri.013G109300.1 | | Potri.013G046300.1 | | Potri.013G148600.1 | | Potri.013G149100.1 | | Potri.013G056400.1 | | Potri.003G144300.1  Potri.011G040300.1  Potri.011G040400.1  Potri.013G056500.1  Potri.013G149200.1  Potri.017G125700.1  Potri.017G125800.1  Potri.017G125900.1  Potri.017G126000.1  Potri.019G036400.1  Potri.019G118800.1  Potri.019G118900.1   | Potri.002G173900.1 | | --- | | Potri.003G155700.1 | | Potri.003G144200.1 | | Potri.003G132000.1 | | Potri.003G114100.1 | | Potri.003G094200.1 | | Potri.003G064600.1 | | Potri.003G219900.1 | | Potri.003G189700.1 | | Potri.009G061500.1 | | Potri.009G096000.1 | | Potri.009G027300.1 | | Potri.009G042600.1 | | Potri.009G044100.1 | | Potri.009G053900.1 | | Potri.011G167600.1 | | Potri.005G224100.1 | | Potri.005G186400.1 | | Potri.005G164900.1 | | Potri.014G022500.1 | | Potri.014G035100.1 | | Potri.014G054700.1 | | Potri.014G081200.1 | | Potri.014G100800.1 | | Potri.014G111200.1 | | Potri.014G117000.1 | | Potri.014G122700.1 | | Potri.009G007100.1 | | Potri.009G134000.1 | | Potri.017G130300.1 | | Potri.017G128900.1 | | Potri.017G112300.1 | | Potri.011G040200.1 | | Potri.011G041600.1 | | Potri.007G048900.1 | | Potri.007G106100.1 | | Potri.007G093900.1 | | Potri.007G076200.1 | | Potri.007G067600.1 | | Potri.013G001000.1 | | Potri.018G127700.1 | | Potri.018G095900.1 | | Potri.016G112300.1 | | Potri.017G017600.1 | | Potri.010G123000.1 | | Potri.010G114000.1 | | Potri.010G093000.1 | | Potri.010G064000.1 | | Potri.010G195000.1 | | Potri.010G174500.1 | | Potri.010G167500.1 | | Potri.010G165700.1 | | Potri.010G149900.1 | | Potri.015G046200.1 | | Potri.015G129100.1 | | Potri.015G143500.1 | | Potri.015G067700.1 | | Potri.015G075600.1 | | Potri.015G077700.1 | | Potri.019G081500.1 | | Potri.019G018400.1 | | Potri.019G118200.1 | | Potri.019G118700.1 | | Potri.019G036300.1 | | Tandem duplication  Tandem duplication  Tandem duplication  Tandem duplication  Tandem duplication  Tandem duplication  Tandem duplication  Tandem duplication  Tandem duplication  Tandem duplication  Tandem duplication  Tandem duplication  Segmental duplication  Segmental duplication  Segmental duplication  Segmental duplication  Segmental duplication  Segmental duplication  Segmental duplication  Segmental duplication  Segmental duplication  Segmental duplication  Segmental duplication  Segmental duplication  Segmental duplication  Segmental duplication  Segmental duplication  Segmental duplication  Segmental duplication  Segmental duplication  Segmental duplication  Segmental duplication  Segmental duplication  Segmental duplication  Segmental duplication  Segmental duplication  Segmental duplication  Segmental duplication  Segmental duplication  Segmental duplication  Segmental duplication  Segmental duplication  Segmental duplication  Segmental duplication  Segmental duplication  Segmental duplication  Segmental duplication  Segmental duplication  Segmental duplication  Segmental duplication  Segmental duplication  Segmental duplication  Segmental duplication  Segmental duplication  Segmental duplication  Segmental duplication  Segmental duplication  Segmental duplication  Segmental duplication  Segmental duplication  Segmental duplication  Segmental duplication  Segmental duplication  Segmental duplication  Segmental duplication  Segmental duplication  Segmental duplication  Segmental duplication  Segmental duplication  Segmental duplication  Segmental duplication  Segmental duplication  Segmental duplication  Segmental duplication  Segmental duplication  Segmental duplication | | 0.228918 | | --- | | 0.097822 | | 0.102022 | | 0.040487 | | 0.494956 | | 0.132342 | | 0.087449 | | 0.083214 | | 0.087469 | | 0.005625 | | 0.302422 | | 0.479981 | | 0.390421 | | 0.097541 | | 0.100263 | | 0.033256 | | 0.040213 | | 0.076348 | | 0.108505 | | 0.073741 | | 0.079209 | | 0.065259 | | 0.089061 | | 0.061707 | | 0.045733 | | 0.111895 | | 0.07551 | | 0.05104 | | 0.090466 | | 0.073237 | | 0.069841 | | 0.072134 | | 0.073404 | | 0.059818 | | 0.058769 | | 0.077167 | | 0.061517 | | 0.088523 | | 0.147181 | | 0.062224 | | 0.051617 | | 0.090151 | | 0.066331 | | 0.082016 | | 0.204355 | | 0.06709 | | 0.101254 | | 0.076249 | | 0.052377 | | 0.038308 | | 0.111392 | | 0.050567 | | 0.079237 | | 0.073206 | | 0.046969 | | 0.092598 | | 0.098936 | | 0.106689 | | 0.061172 | | 0.046617 | | 0.046573 | | 0.105644 | | 0.113502 | | 0.085206 | | 0.056763 | | 0.066157 | | 0.075124 | | 0.122432 | | 0.087353 | | 0.189478 | | 0.105412 | | 0.084816 | | 0.051733 | | 0.081675 | | 0.099354 | | 0.146645 | | | 0.587762 | | --- | | 0.151494 | | 0.258509 | | 0.12377 | | 5.125582 | | 0.169112 | | 0.152938 | | 0.200409 | | 0.161809 | | 0.014207 | | 1.00423 | | 1.547126 | | 1.885962 | | 0.286183 | | 0.247865 | | 0.23113 | | 0.314004 | | 0.231195 | | 0.368628 | | 0.2379 | | 0.168838 | | 0.193809 | | 0.350315 | | 0.264659 | | 0.251874 | | 0.277164 | | 0.237484 | | 0.319528 | | 0.263094 | | 0.168143 | | 0.174117 | | 0.383119 | | 0.366423 | | 0.282854 | | 0.22071 | | 0.343731 | | 0.248778 | | 0.415387 | | 0.402033 | | 0.23668 | | 0.421194 | | 0.249065 | | 0.232754 | | 0.399871 | | 0.556374 | | 0.357031 | | 0.450698 | | 0.205166 | | 0.198519 | | 0.220215 | | 0.299411 | | 0.189499 | | 0.24866 | | 0.198826 | | 0.188313 | | 0.274461 | | 0.215663 | | 0.320174 | | 0.214292 | | 0.260657 | | 0.196267 | | 0.308639 | | 0.234982 | | 0.293171 | | 0.226498 | | 0.323378 | | 0.322485 | | 0.324305 | | 0.244603 | | 0.422482 | | 0.302381 | | 0.32558 | | 0.235004 | | 0.21426 | | 0.241626 | | 0.457349 | | | 0.389475 | | --- | | 0.645711 | | 0.394654 | | 0.327114 | | 0.096566 | | 0.782572 | | 0.571791 | | 0.415221 | | 0.540571 | | 0.395897 | | 0.301148 | | 0.310241 | | 0.207014 | | 0.340835 | | 0.404507 | | 0.143886 | | 0.128066 | | 0.330234 | | 0.294347 | | 0.309967 | | 0.469143 | | 0.336716 | | 0.254231 | | 0.233158 | | 0.18157 | | 0.403714 | | 0.317958 | | 0.159736 | | 0.343855 | | 0.435566 | | 0.401118 | | 0.188282 | | 0.200324 | | 0.211479 | | 0.266274 | | 0.224498 | | 0.247275 | | 0.213109 | | 0.366092 | | 0.262903 | | 0.122549 | | 0.361959 | | 0.284985 | | 0.205107 | | 0.367299 | | 0.187912 | | 0.22466 | | 0.371645 | | 0.26384 | | 0.173957 | | 0.372037 | | 0.266845 | | 0.318656 | | 0.368194 | | 0.249421 | | 0.33738 | | 0.458752 | | 0.333223 | | 0.285461 | | 0.178844 | | 0.237293 | | 0.342288 | | 0.483022 | | 0.290637 | | 0.250612 | | 0.20458 | | 0.232953 | | 0.377522 | | 0.357122 | | 0.448488 | | 0.348605 | | 0.260509 | | 0.220139 | | 0.381194 | | 0.411187 | | 0.320642 | | Purifying selection  Purifying selection  Purifying selection  Purifying selection  Purifying selection  Purifying selection  Purifying selection  Purifying selection  Purifying selection  Purifying selection  Purifying selection  Purifying selection  Purifying selection  Purifying selection  Purifying selection  Purifying selection  Purifying selection  Purifying selection  Purifying selection  Purifying selection  Purifying selection  Purifying selection  Purifying selection  Purifying selection  Purifying selection  Purifying selection  Purifying selection  Purifying selection  Purifying selection  Purifying selection  Purifying selection  Purifying selection  Purifying selection  Purifying selection  Purifying selection  Purifying selection  Purifying selection  Purifying selection  Purifying selection  Purifying selection  Purifying selection  Purifying selection  Purifying selection  Purifying selection  Purifying selection  Purifying selection  Purifying selection  Purifying selection  Purifying selection  Purifying selection  Purifying selection  Purifying selection  Purifying selection  Purifying selection  Purifying selection  Purifying selection  Purifying selection  Purifying selection  Purifying selection  Purifying selection  Purifying selection  Purifying selection  Purifying selection  Purifying selection  Purifying selection  Purifying selection  Purifying selection  Purifying selection  Purifying selection  Purifying selection  Purifying selection  Purifying selection  Purifying selection  Purifying selection  Purifying selection  Purifying selection |
