# Supplementary material for: Characterization of the Poplar R2R3-MYB Gene Family and Over-Expression of PsnMYB108 Confers Salt Tolerance in Transgenic Tobacco
Source: Front Plant Sci. 2020 Oct 16;11:571881. doi: 10.3389/fpls.2020.571881 (PMC7596293; doi:10.3389/fpls.2020.571881)
Supplement: Supplementary Table 3 — List of syntenic gene pairs. [file Table_3.DOC]

Syntenic gene pairs

| Gene ID | Gene ID | Ka | Ks | Ka/Ks | | Selection pressure |
| --- | --- | --- | --- | --- | --- | --- |
| | Potri.001G099800.1 | | --- | | Potri.001G118800.1 | | Potri.001G118800.1 | | Potri.001G248800.1 | | Potri.001G250000.1 | | Potri.002G185900.1 | | Potri.002G191800.1 | | Potri.002G198100.1 | | Potri.003G114100.1 | | Potri.003G132000.1 | | Potri.005G142600.1 | | Potri.006G097300.1 | | Potri.006G123400.1 | | Potri.006G170800.1 | | Potri.007G048900.1 | | Potri.008G062700.1 | | Potri.008G062700.1 | | Potri.008G064200.1 | | Potri.008G064200.1 | | Potri.008G088000.1 | | Potri.008G088000.1 | | Potri.008G128500.1 | | Potri.009G042600.1 | | Potri.009G044100.1 | | Potri.009G134000.1 | | Potri.009G134000.1 | | Potri.009G134000.1 | | Potri.010G167500.1 | | Potri.010G195000.1 | | Potri.010G195000.1 | | Potri.011G125900.1 | | Potri.014G111200.1 | | Potri.014G117000.1 | | Potri.014G122700.1 | | Potri.017G082500.1 | | Potri.017G085200.1 | | Potri.017G085200.1 | | Potri.017G086300.1 | | Potri.017G099500.1 | | Potri.018G095900.1 | | | AT1G63910.1 | | --- | | AT4G12350.1 | | AT4G22680.1 | | AT5G58900.1 | | AT5G58850.1 | | AT4G01680.2 | | AT2G47190.1 | | AT2G47460.1 | | AT4G12350.1 | | AT1G63910.1 | | AT5G67300.1 | | AT2G38090.1 | | AT2G36890.1 | | LOC_Os02g54520.1 | | AT5G67300.1 | | AT3G55730.1 | | AT2G39880.1 | | AT5G05790.1 | | AT3G11280.1 | | AT1G26780.2 | | AT1G69560.1 | | AT1G71030.1 | | AT5G58900.1 | | AT5G58850.1 | | LOC_Os09g36730.1 | | AT4G38620.1 | | AT4G34990.1 | | AT1G69560.1 | | AT2G39880.1 | | AT3G55730.1 | | AT5G54230.1 | | AT4G01680.2 | | AT2G47190.1 | | AT2G47460.1 | | AT3G28910.1 | | AT5G39700.1 | | AT3G29020.2 | | AT5G15310.1 | | AT3G30210.1 | | AT5G57620.1 | | | | 0.309475 | | --- | | 0.271176 | | 0.292942 | | 0.253812 | | 0.497764 | | 0.368528 | | 0.434377 | | 0.586808 | | 0.278704 | | 0.287911 | | 0.324456 | | 0.234663 | | 0.428286 | | 0.520128 | | 0.320092 | | 0.429274 | | 0.537576 | | 0.410043 | | 0.34052 | | 0.525386 | | 0.521255 | | 0.57077 | | 0.287179 | | 0.575637 | | 0.235243 | | 0.273186 | | 0.31955 | | 0.547089 | | 0.530214 | | 0.43945 | | 0.461475 | | 0.338943 | | 0.441908 | | 0.574697 | | 0.276157 | | 0.470249 | | 0.606483 | | 0.22346 | | 0.26461 | | 0.350937 | | | --- | --- | --- | --- | --- | --- | --- | --- | --- | --- | --- | --- | --- | --- | --- | --- | --- | --- | --- | --- | --- | --- | --- | --- | --- | --- | --- | --- | --- | --- | --- | --- | --- | --- | --- | --- | --- | --- | --- | --- | --- | | | NaN | | --- | | NaN | | NaN | | NaN | | NaN | | 2.625869 | | NaN | | NaN | | NaN | | 4.791421 | | 2.131562 | | NaN | | 1.261884 | | NaN | | 2.117354 | | 1.623541 | | 1.599546 | | NaN | | NaN | | 3.312379 | | 1.781547 | | 1.531075 | | NaN | | NaN | | NaN | | 1.82521 | | 1.989979 | | 3.78425 | | 1.50756 | | 1.327745 | | NaN | | NaN | | NaN | | NaN | | 2.190577 | | 3.217136 | | 1.389487 | | 2.07846 | | NaN | | 3.120282 | | | NaN | | --- | | NaN | | NaN | | NaN | | NaN | | 0.140345 | | NaN | | NaN | | NaN | | 0.060089 | | 0.152215 | | NaN | | 0.339402 | | NaN | | 0.151176 | | 0.264406 | | 0.33608 | | NaN | | NaN | | 0.158613 | | 0.292586 | | 0.37279 | | NaN | | NaN | | NaN | | 0.149674 | | 0.160579 | | 0.14457 | | 0.351703 | | 0.330975 | | NaN | | NaN | | NaN | | NaN | | 0.126066 | | 0.14617 | | 0.436479 | | 0.107512 | | NaN | | 0.11247 | | NaN  NaN  NaN  NaN  NaN  Purifying selection  NaN  NaN  NaN  Purifying selection  Purifying selection  NaN  Purifying selection  NaN  Purifying selection  Purifying selection  Purifying selection  NaN  NaN  Purifying selection  Purifying selection  Purifying selection  NaN  NaN  NaN  Purifying selection  Purifying selection  Purifying selection  Purifying selection  Purifying selection  NaN  NaN  NaN  NaN  Purifying selection  Purifying selection  Purifying selection  Purifying selection  NaN  Purifying selection | |
