# Supplementary material for: Characterization of the Poplar R2R3-MYB Gene Family and Over-Expression of PsnMYB108 Confers Salt Tolerance in Transgenic Tobacco
Source: Front Plant Sci. 2020 Oct 16;11:571881. doi: 10.3389/fpls.2020.571881 (PMC7596293; doi:10.3389/fpls.2020.571881)
Supplement: Supplementary Table 4 — Annotations of differentially expressed genes. [file Table_4.DOC]

Annotations of differentially expressed genes

|  | ID | Best-hit-arabi-name |
| --- | --- | --- |
| Gene differently expressed in leaf, root, and stem without salt treatment | | Potri.019G045900.1 | | --- | | Potri.017G086300.1 | | Potri.017G082500.1 | | Potri.006G122100.1 | | Potri.009G134000.1 | | Potri.006G123400.1 | | Potri.014G035100.1 | | Potri.008G166700.1 | | | AT1G08810.1 | | --- | | AT5G15310.2 | | AT3G47600.1 | | AT3G53200.1 | | AT4G38620.1 | | AT5G57620.1 | | AT4G37260.1 | | AT3G23250.1 | |
| Differential expression genes in response to salinity | | Potri.013G001000.1 | | --- | | Potri.005G001600.1 | | Potri.017G112300.1 | | Potri.012G080400.1 | | Potri.008G064200.1 | | Potri.012G060300.1 | | Potri.009G134000.1 | | Potri.004G088100.1 | | Potri.019G045900.1 | | Potri.014G111200.1 | | Potri.012G084100.1 | | Potri.002G185900.1 | | Potri.016G112300.1 | | Potri.007G048900.1 | | Potri.009G027300.1 | | Potri.010G240800.1 | | Potri.019G081500.1 | | Potri.T011400.1 | | Potri.008G122100.1 | | Potri.004G033100.1 | | Potri.002G122600.1 | | Potri.008G101400.1 | | Potri.011G041600.1 | | Potri.010G123000.1 | | Potri.017G082500.1 | | Potri.001G235500.1 | | Potri.009G096000.1 | | Potri.010G149900.1 | | Potri.015G046200.1 | | Potri.004G026600.1 | | Potri.005G112000.1 | | Potri.018G005300.1 | | Potri.006G275900.1 | | Potri.004G102600.1 | | Potri.013G067000.1 | | Potri.007G007900.1 | | Potri.012G055600.1 | | Potri.001G408700.1 | | Potri.015G033600.1 | | Potri.009G007100.1 | | Potri.002G173900.1 | | Potri.009G042600.1 | | Potri.008G128500.1 | | Potri.003G144300.1 | | Potri.015G082700.1 | | Potri.005G074500.1 | | Potri.010G093000.1 | | Potri.001G336700.1 | | Potri.006G097300.1 | | Potri.005G164900.1 | | Potri.004G138000.1 | | Potri.006G123400.1 | | Potri.004G174400.1 | | Potri.001G005100.1 | | Potri.013G046300.1 | | Potri.014G035100.1 | | Potri.012G140500.1 | | Potri.018G095900.1 | | Potri.003G189700.1 | | Potri.006G221800.1 | | Potri.007G067600.1 | | Potri.005G096600.1 | | Potri.015G129100.1 | | Potri.019G036400.1 | | Potri.017G099500.1 | | Potri.001G219100.1 | | Potri.005G224100.1 | | Potri.005G063200.1 | | Potri.014G100800.1 | | Potri.004G086300.1 | | Potri.002G038500.1 | | Potri.013G149200.1 | | Potri.008G166700.1 | | Potri.010G064000.1 | | Potri.014G117000.1 | | Potri.011G125900.1 | | Potri.013G149100.1 | | Potri.002G191800.1 | | Potri.008G089200.1 | | Potri.017G130300.1 | | Potri.019G118700.1 | | Potri.011G040300.1 | | Potri.014G054700.1 | | Potri.001G347200.1 | | Potri.017G075000.1 | | | AT1G09540.1 | | --- | | AT1G09540.1 | | AT2G37630.1 | | AT5G14750.1 | | AT5G05790.1 | | AT1G49010.1 | | AT4G38620.1 | | AT4G09460.1 | | AT1G08810.1 | | AT4G01680.1 | | AT5G26660.1 | | AT4G01680.1 | | AT2G38090.1 | | AT4G37260.1 | | AT3G46130.1 | | AT5G04760.1 | | AT1G22640.1 | | AT4G38620.1 | | AT1G68320.1 | | AT4G21440.1 | | AT4G37260.1 | | AT3G06490.1 | | AT4G21440.1 | | AT1G68320.1 | | AT3G47600.1 | | AT3G46130.1 | | AT4G37260.1 | | AT3G06490.1 | | AT1G25340.1 | | AT4G21440.1 | | AT4G38620.1 | | AT1G22640.1 | | AT1G22640.1 | | AT2G37630.1 | | AT5G17800.1 | | AT3G49690.1 | | AT1G68320.1 | | AT4G21440.1 | | AT1G17950.1 | | AT5G57620.1 | | AT1G22640.1 | | AT5G58900.1 | | AT4G38620.1 | | AT2G47460.1 | | AT1G57560.1 | | AT1G34670.1 | | AT2G02820.2 | | AT5G14340.1 | | AT2G38090.1 | | AT1G34670.1 | | AT4G38620.1 | | AT5G57620.1 | | AT4G38620.1 | | AT3G13540.1 | | AT1G09770.1 | | AT4G37260.1 | | AT5G52260.1 | | AT5G57620.1 | | AT5G06100.2 | | AT4G38620.1 | | AT1G79180.1 | | AT1G79180.1 | | AT4G22680.1 | | AT3G13540.1 | | AT3G30210.1 | | AT5G04760.1 | | AT3G23250.1 | | AT4G33450.1 | | AT2G16720.1 | | AT5G16600.1 | | AT2G31180.1 | | AT2G31180.1 | | AT3G23250.1 | | AT4G13480.1 | | AT2G47190.1 | | AT4G21440.1 | | AT3G23250.1 | | AT2G47190.1 | | AT3G01140.1 | | AT5G16600.1 | | AT5G16770.2 | | AT4G38620.1 | | AT3G60460.1 | | AT3G27785.1 | | AT3G28470.1 | |
